# Exploring the neuroprotective potential of Nrf2-pathway activators against annonacin toxicity

**DOI:** 10.1038/s41598-024-70837-1

**Published:** 2024-08-29

**Authors:** Márcia F. D. Costa, Thomas W. Rösler, Günter U. Höglinger

**Affiliations:** 1 German Center for Neurodegenerative Diseases (DZNE), Munich, Germany; 2 Department of Neurology, Hannover Medical School, Hannover, Germany; 3 Munich Cluster for Systems Neurology (SyNergy), Munich, Germany; 4 Department of Neurology, LMU Hospital, Ludwig-Maximilians-University (LMU), Munich, Germany; 5 Laboratory of Pharmacology, Department of Diagnostics and Public Health, University of Verona, Verona, Italy

**Keywords:** Neurodegenerative diseases, Neurodegeneration, Neurodegeneration

## Abstract

Modulation of the Nrf2 pathway, a master regulator of the antioxidant response and cellular metabolism, has been suggested as a promising therapeutic strategy in tauopathies, a heterogeneous group of neurodegenerative disorders characterized by intracellular proteinaceous inclusions of abnormally phosphorylated tau. Here, we explored the neuroprotective potential of different Nrf2-pathway activators in human immortalized dopaminergic neurons against annonacin-induced toxicity, a mitochondrial inhibitor associated with a PSP-like syndrome and capable of mimicking tauopathy-like features. Interestingly, we observed heterogenous and compound-dependent neuroprotective effects among the different Nrf2-pathway activators. With the exception of Fyn inhibitors, all the selected Nrf2-pathway activators improved cell viability and the oxidative status, and reduced the annonacin-induced tau hyperphosphorylation and neurite degeneration, particularly the p62-activators. However, improvement of the impaired mitochondrial function was only observed by the Bach-1 inhibitor. Surprisingly, we found evidence that ezetimibe, an approved drug for hypercholesterolemia, prevents the transcriptional upregulation of 4R-tau triggered by annonacin insult. Overall, our results suggest that the neuroprotective effects of the Nrf2-pathway activators against annonacin toxicity may rely on the specific mechanism of action, intrinsic to each compound, and possibly on the concomitant modulation of additional signaling pathways. Further research will be needed to fully understand how synergistic modulation of metabolic adaptation and cell survival can be exploit to develop new therapeutical strategies for tauopathies and eventually other neurodegenerative diseases.

## Introduction

Oxidative and metabolic stresses are commonly present in the aging brain and have been strongly implicated in tauopathies, as well as in other neurodegenerative disorders^1,2^. A major regulator of the cellular antioxidant response is the transcription factor Nrf2 (nuclear factor erythroid-2-related factor 2). Under homeostatic conditions, Nrf2 is repressed by KEAP1 (kelch-like ECH-associated protein 1), a redox sensor that promotes Nrf2 ubiquitination and consequent proteasomal degradation^3^. In response to oxidative stress, KEAP1 is oxidized and dissociates from Nrf2. After phosphorylation at Ser-40, Nrf2 is translocated into the nucleus and exerts its transcriptional activity^4^. Alternatively, the transcriptional activity of Nrf2 is promoted non-canonically by the autophagy receptor p62/SQSTM1 that binds to KEAP1 and targets it for degradation^5^. Furthermore, Nrf2 participates in the signal transduction of the PERK (pancreatic endoplasmic reticulum kinase) pathway, one of the sensor proteins of endoplasmic reticulum stress and a regulator of the unfolded protein response^6^. Dysfunctions in the Nrf2 pathway have been associated with the pathophysiological process of tauopathies^7–10^. Immunohistochemistry analysis of brains from AD (Alzheimer´s disease) patients suggests a blockage of Nrf2 nuclear translocation and, consequently, lower Nrf2 transcriptional activity^7^. Conversely, activation of the Nrf2 pathway was associated with an attenuation of oxidative stress, lower GSK3β activity, a major kinase responsible for tau phosphorylation, and amelioration of locomotor and cognitive functions in tauopathy mouse models^11–13^. Previously, we found that genetic variants in the PERK encoding gene, *EIF2AK3,* increase the risk of development of PSP (progressive supernuclear palsy) tauopathy^14^. Further investigation using in vitro and in vivo models of tauopathies supported the idea that the protectiveness of PERK activation was predominantly dependent on the PERK-Nrf2 axis, since evidence suggested a downregulation of the PERK-eIF2a axis in the context of tauopathies^15^.

Activation of the Nrf2 pathway can be achieved by promoting the release of Nrf2 from its cytosolic repressor KEAP1, using non-electrophilic Nrf2-activators (e.g. Ki 696), which weaken KEAP1-Nrf2 binding^16^, or by promoting the proteosomal degradation of KEAP1 via p62-activators (e.g. licochalcone A or ezetimibe)^17,18^. Alternatively, Nrf2-transcriptional activity can be promoted with Bach1 inhibitors (e.g. HPP-D), a repressor of Nrf2-transcriptional activity^19^, or by Fyn kinase inhibitors (e.g. PP2 or saracatinib), which prevent Nrf2-nuclear exportation^20,21^. So far, the neuroprotective outcomes of theses diverse strategies to activate the Nrf2-pathway in the context of tau dysfunction have not been compared. Therefore, we aimed to explore if mechanistically diverse Nrf2-pathway activators exhibit similar neuroprotective potentials against annonacin toxicity, a mitochondrial complex I inhibitor that induces features of tau pathology, such as energetic impairment, changes in tau expression, phosphorylation and location, and is therefore considered a pharmacological model of tauopathy^22,23^.

## Results

### The neuroprotective effects of the Nrf2-pathway activators

**Fig. 1:**
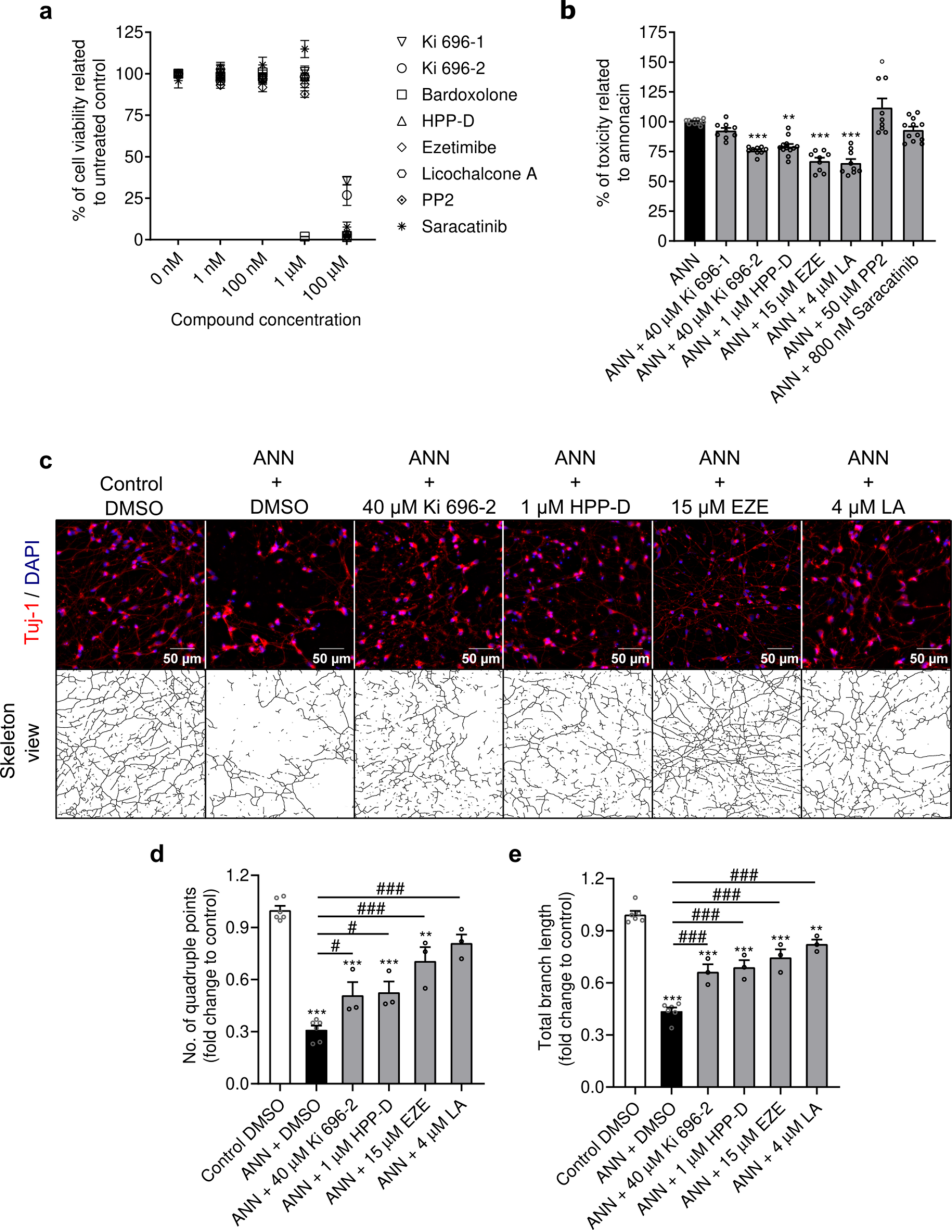
Effects of different Nrf2-pathway activators against annonacin toxicity. (**a**) Toxicity of different Nrf2-pathway activators. LUHMES cells were treated with different concentrations of Nrf2-pathway activators in differentiation medium for 96 h. Cell viability was measured by calcein fluorescence and reported as a percentage relative to the untreated control. Data are plotted as mean ± SEM from 3 independent experiments. (**b**) Reduction of annonacin toxicity by different Nrf2-pathway activators. LUHMES cells were treated with previously established concentrations of different Nrf2-pathway activators in the presence of 20 nM annonacin (ANN), in glucose-reduced medium for 48 h. Cell viability results of the most protective or highest non-toxic concentration of each Nrf2-pathway activator (as shown in Supplementary Fig. S1 online) were expressed as a percentage of annonacin toxicity (ezetimibe: EZE; Licochalcone A: LA). Data are plotted as mean ± SEM. **p < 0.01 and ***p < 0.001 vs. annonacin-DMSO condition (non-parametric Kruskal–Wallis’s test; N = 3). (**c**) Protective effects of the Nrf2-pathway activators against annonacin-induced neurite degeneration. The upper row shows representative immunofluorescence images with 200 × magnification of Tuj-1 antigen (red) with DAPI-counterstained cell nuclei (blue) for assessment of neurite morphology. For analysis, images were binarized and skeletonized. The full skeleton with soma subtraction is shown in the bottom row. Scale bar: 50 µm. The number of quadruple points (**d**) and total branch length (**e**) are plotted as mean ± SEM. **p < 0.01 and ***p < 0.001 vs. control. ^#^p < 0.05 and ^###^p < 0.001 vs. annonacin (One-way ANOVA with Sidak’s post hoc test; N = 3).

We started by measuring the impact of increased concentrations of different Nrf2-pathway activators on the viability of differentiated dopaminergic-like LUHMES (lund human mesencephalic) neuronal cells (Fig. 1a). We found that the majority of the Nrf2-pathway activators were well-tolerated in a wide concentration range, from 1 nM to 100 μM, with only one compound, bardoxolone, being extremely neurotoxic at concentrations above the nanomolar range. For this reason, we decided to withdraw bardoxolone from our study. Next, we titrated annonacin to select a suitable toxic concentration for the pharmacological experiments (Supplementary Fig. S1a online). We defined 20 nM annonacin as an appropriate concentration for subsequent experiments, as it provided a significant reduction of cell viability (p < 0.0001) to approximately 50% of the untreated condition (0 nM). The protective effects of the different Nrf2-pathway activators were then assessed in terms of cell viability, with increased compound concentrations (ranging from 500 nM to 50 μM), in the presence of 20 nM of annonacin, in glucose-reduced medium (Supplementary Fig. S1b online). To compare the protection efficacy of the different Nrf2-pathway activators, we defined the maximum toxicity (100%) as the condition where cells were intoxicated with annonacin and did not receive any test compound (annonacin (ANN) condition). Then, we selected the most protective concentration for each Nrf2-pathway activator, or the highest non-toxic concentration in the case of the non-protective compounds Ki 696–1 and PP2, and expressed their effects on cell viability as a reduction or gain of cellular toxicity relative to the annonacin condition (Fig. 1b). We found no statistical differences between the annonacin condition and cells treated with Ki 696–1 or Fyn kinase inhibitors, PP2 and saracatinib (p > 0.999). The treatment with 40 μM Ki 696–2 and 1 μM HPP-D led to a significant reduction of annonacin toxicity by 24.2% (p = 0.0003) and 21% (p = 0.0014), respectively. The p62-activators, ezetimibe and licochalcone A, exhibited the strongest protection, attenuating the annonacin-induced toxicity by 33% (p < 0.0001) and 34.6% (p < 0.0001), respectively. Based on these results, we selected 40 μM Ki 696–2, 1 μM HPP-D, 15 μM ezetimibe, and 4 μM licochalcone A as protective concentrations against annonacin toxicity, to be used in subsequent experiments. The Ki 696-1 and the Fyn kinase inhibitors PP2 and saracatinib were excluded from further analysis, due to the lack of evidence supporting a neuroprotective role under the paradigm of this study.

The protectiveness of the selected Nrf2-pathway activators was further confirmed by assessing their capacity to prevent annonacin-induced neurite degeneration (Fig. 1c). In cells exposed to annonacin (ANN condition), we observed a drastic and significant reduction of the number of quadruple points (i.e. junction points formed by four branches of the neuronal arbor) to 0.31-fold over control (p < 0.0001) (Fig. 1d), and a reduction to 0.44-fold over control of the total branch length (p < 0.0001) (Fig. 1e). All the selected Nrf2-pathway activators had a positive impact on attenuating the annonacin-induced neurite degeneration, contributing to a higher complexity of the neural arborization, as translated by the number of quadruple points. These effects ranged from mild in the case of the compounds Ki 696–2 (p < 0.0001 vs. control; p = 0.049 vs. annonacin) and HPP-D (p < 0.0001 vs. control; p = 0.028 vs. annonacin), both yielding approximately 0.50-fold over control, to a much more pronounced impact of ezetimibe and licochalcone A, which led to a 0.71-fold (p = 0.002 vs. control; p < 0.0001 vs. annonacin) and 0.81-fold (p = 0.069 vs. control; p < 0.0001 vs. annonacin) over control, respectively (Fig. 1d). The protective Nrf2-pathway activators also contributed to significantly denser neural arborizations compared with the annonacin condition (p < 0.0001 for Ki 696–2 and HPP-D; p = 0.0001 for ezetimibe and p = 0.007 for licochalcone A vs. control; p = 0.0004 for Ki 696–2, p = 0.0001 for HPP-D and p < 0.0001 for ezetimibe and licochalcone A vs. annonacin), as given by the total branch length (Fig. 1e). Overall, treatment with the selected Nrf2-pathway activators, particularly with the p62-activators, ezetimibe and licochalcone A, limited the neuronal death and neurite degeneration induced by annonacin.

### Effects of the Nrf2-pathway activators against mitochondrial dysfunction

**Fig. 2:**
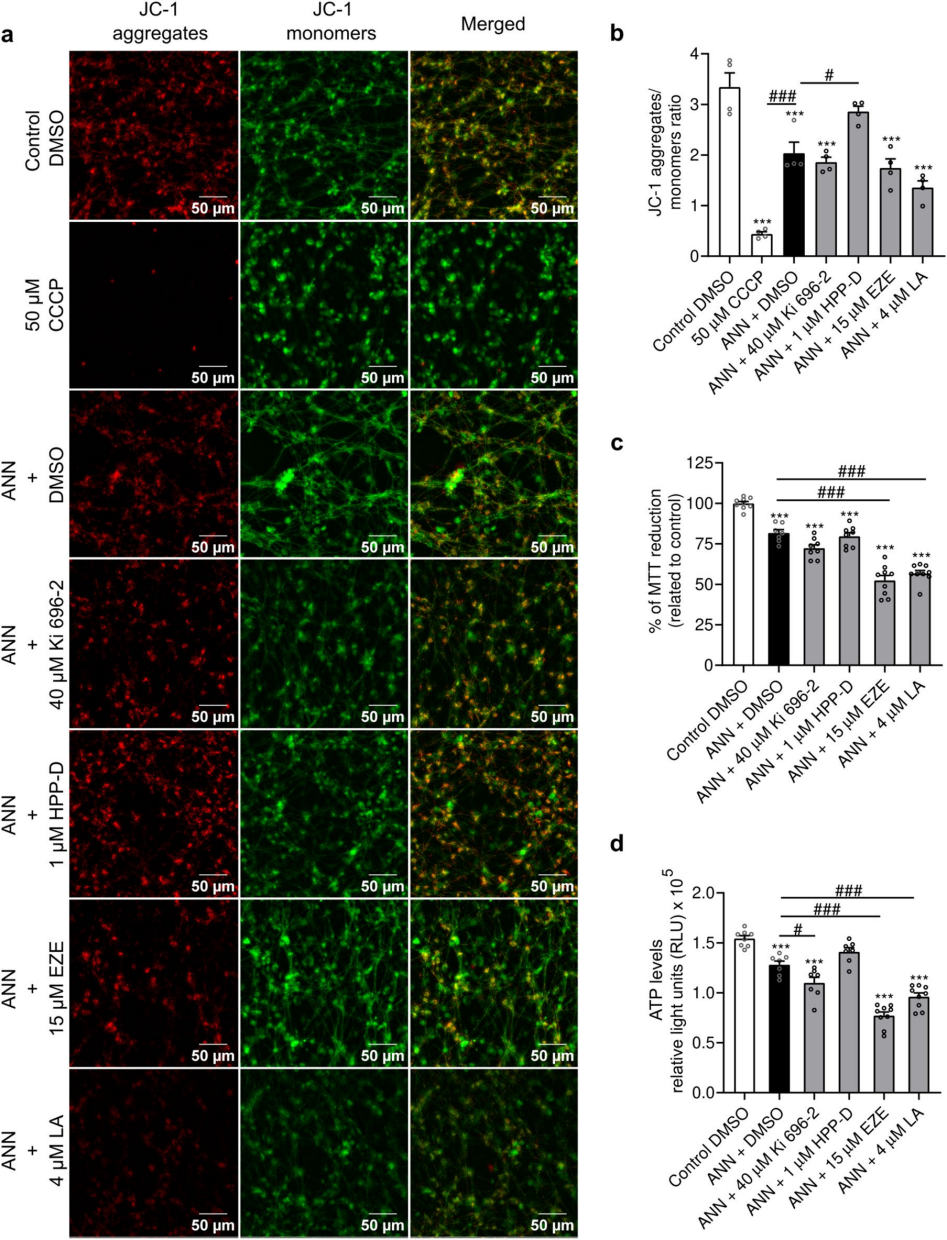
HPP-D rescued mitochondria from annonacin-induced depolarization. (**a**) LUHMES cells were treated with protective concentrations of different Nrf2-pathway activators in the presence of 20 nM annonacin (ANN), in glucose-reduced medium for 24 h. JC-1 probe was used to assess mitochondrial membrane potential. The JC-1 monomeric form spontaneously aggregates in polarized mitochondria. A reduction in the fluorescence intensity of JC-1 aggregates is indicative of mitochondrial depolarization. Representative fluorescence microscopy images with 200 × magnification of JC-1-positive mitochondrial aggregates (red) and monomers (green), and respective merged channels are provided. Scale bar: 50 µm. (**b**) Quantification of the fluorescence intensity of JC-1 aggregates and monomers expressed as an aggregates/monomers ratio (ezetimibe: EZE; Licochalcone A: LA). Data are plotted as mean ± SEM (One-way ANOVA with Sidak’s post hoc test; N = 4). (**c**) Mitochondrial activity was estimated by reduction of MTT. Colorimetric absorbance values were expressed as a percentage of untreated control. Data are plotted as mean ± SEM (Brown-Forsythe ANOVA test with Dunnett’s T3 post hoc test; N = 3). (**d**) The ATP levels were determined with ViaLight™ plus kit. The luminescence values were expressed as RLU (relative light units). Data are plotted as mean ± SEM (One-way ANOVA with Sidak’s post hoc test; N = 3). ***p < 0.001 vs. control. ^#^*p* < 0.05 and ^###^*p* < 0.001 vs. annonacin.

As an inhibitor of mitochondrial complex I, annonacin is known for impairing cellular energy metabolism. We aimed to understand if the protectiveness of Nrf2-pathway activators was associated with a lessening of the annonacin-induced mitochondrial dysfunction. For this purpose, we assessed the ΔψM (mitochondrial membrane potential), a key indicator of mitochondrial activity. In healthy cells, the high ΔψM induces spontaneous aggregation of JC-1, while in unhealthy or apoptotic cells, the low ΔψM retains JC-1 in the monomeric form. As anticipated, in control cells, the fluorescence intensity of JC-1 aggregates was considerably higher than that of the monomeric form (Fig. 2a,b). Treatment with the mitochondrial oxidative phosphorylation uncoupler, CCCP, drastically reduced the presence of JC-1 aggregates (p < 0.0001 vs. control) (Fig. 2a,b), thus suggesting a strong mitochondrial membrane depolarization. Likewise, although more moderate, annonacin induced a significant mitochondrial depolarization (p = 0.0002 vs. control and p < 0.0001 vs. CCCP). Interestingly, we observed that only HPP-D was able to rescue the ΔψM from annonacin-induced depolarization (p = 0.027 vs. annonacin) to values no longer statistically different from control (p = 0.465). In order to understand if this rescue was associated with an improvement of mitochondrial function, we estimated the mitochondrial activity by MTT assay and assessed the intracellular ATP levels. Upon a 24 h treatment with annonacin, the amount of reduced-MTT decreased by 20% when compared with healthy control cells (p = 0.0003) (Fig. 2c). Concomitant treatment with Ki 696–2 (p < 0.0001 vs. control and p = 0.072 vs. annonacin), ezetimibe and licochalcone A (p < 0.0001 vs. control and annonacin), further decreased the amount of MTT reduction. This decrease in MTT reduction was followed by lower intracellular ATP levels (p < 0.0001 for Ki 696–2, ezetimibe and licochalcone A vs. control; p = 0.0398 for Ki 696–2 and p < 0.0001 for ezetimibe and licochalcone A vs. annonacin) (Fig. 2d), thus correlating with the changes in the ΔψM. Importantly, the protective effects of HPP-D against mitochondrial depolarization led to intracellular ATP levels no longer statistically different from healthy control cells (p = 0.217), thus suggesting that HPP-D contributed to reducing annonacin-induced mitochondrial dysfunction.

### Nrf2-pathway activators attenuate the oxidative burden

**Fig. 3:**
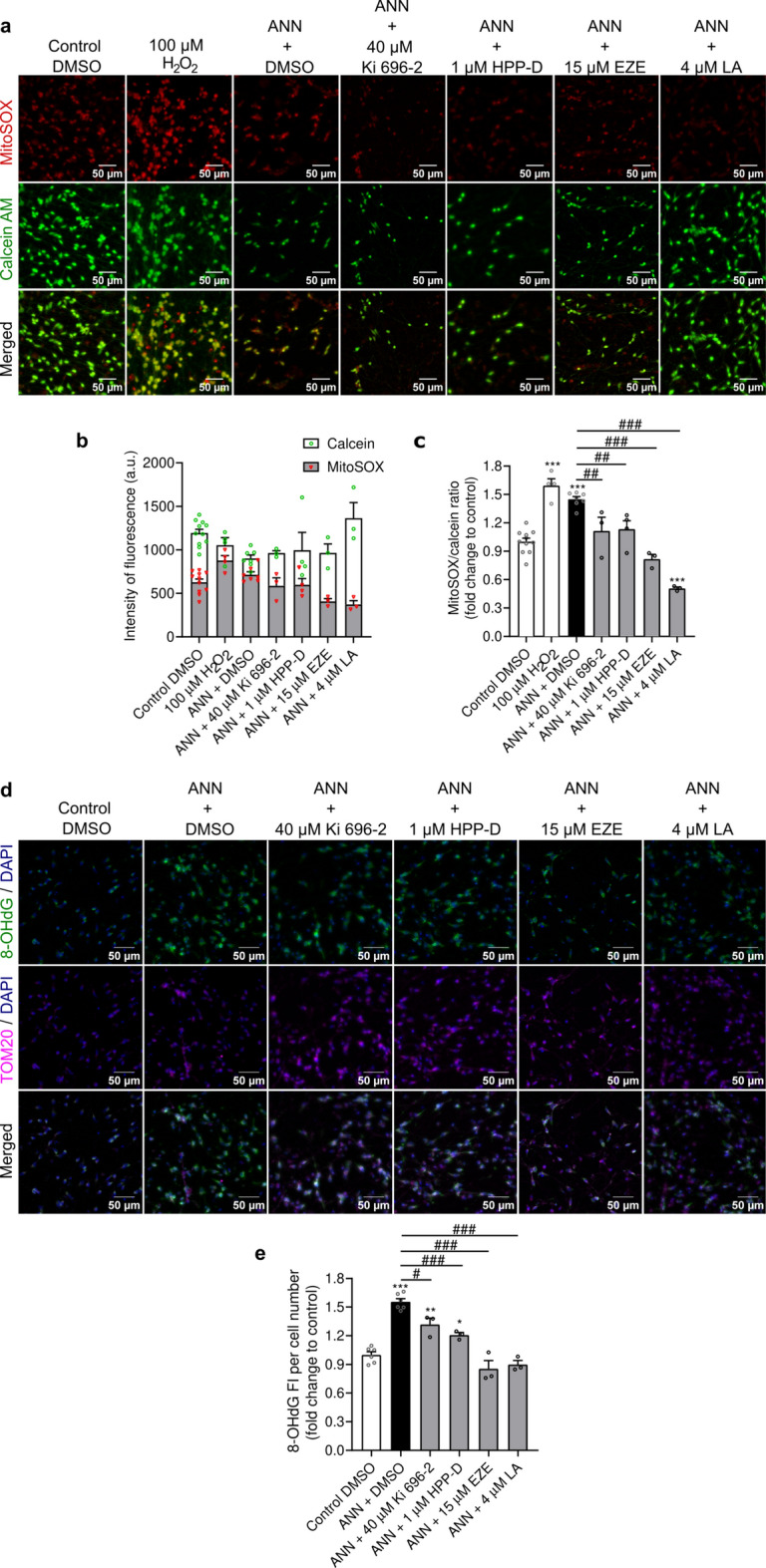
Nrf2-pathway activators reduced annonacin-induced mitochondrial oxidative stress and diminished the presence of the oxidative DNA damage marker 8-OHdG. (**a**) LUHMES cells were treated with protective concentrations of different Nrf2-pathway activators in the presence of 20 nM annonacin (ANN), in glucose-reduced medium for 48 h, and co-stained with the mitochondrial superoxide probe MitoSOX (red) and the cell viability indicator calcein AM (green). For the positive-control, cells were incubated for 2 h with 100 µM H_2_O_2_. Representative images with 200 × magnification of the individualized and merged channels are provided. Scale bar: 50 µm. (**b**) Quantification of the fluorescence intensity of MitoSOX and calcein AM described in (**a**) (ezetimibe: EZE; Licochalcone A: LA). Data are plotted as mean ± SEM. (**c**) MitoSOX fluorescence intensity normalized to calcein AM fluorescence intensity (MitoSOX/calcein ratio) measured in (**b**) expressed as fold change relative to control. Data are plotted as mean ± SEM (One-way ANOVA with Sidak’s post hoc test; N = 3). (**d**) Representative immunofluorescence images with 200 × magnification of the oxidative DNA damage marker 8-OHdG (8-hydroxyguanosine) (green), and the mitochondrial protein TOM20 (magenta), with DAPI-counterstained cell nuclei (blue), and respective merged channels are provided. Scale bar: 50 µm. (**e**) Quantification of the fluorescence intensity (FI) of 8-OHdG normalized to cell number described in (**d**), expressed as fold change relative to control. Data are plotted as mean ± SEM (One-way ANOVA with Sidak’s post hoc test; N = 3). *p < 0.05, **p < 0.01 and ***p < 0.001 vs. control. ^#^*p* < 0.05, ^##^*p* < 0.01 and ^###^*p* < 0.001 vs. annonacin.

Mitochondrial dysfunctions are well-known sources of oxidative stress therefore we investigated the levels of mitochondrial oxidative stress by detecting the presence of mitochondrial superoxide with the fluorescence probe MitoSOX (Fig. 3a). Short incubation with H_2_O_2_, used as a positive control, increased the MitoSOX fluorescence intensity without greatly impairing the cell viability (Fig. 3b), demonstrating that MitoSOX was indeed reporting the presence of superoxide. Although we observed no major changes in MitoSOX fluorescence intensity after exposure to annonacin (Fig. 3b), the proportion of MitoSOX reactivity to the cell viability, inferred by calcein fluorescence, was significantly increased in annonacin-treated cells (p < 0.0001) and comparable to the H_2_O_2_-treated cells (Fig. 3c). This finding suggested that annonacin promoted mitochondrial oxidative stress. We then assessed if the Nrf2-pathway activators could lessen the oxidative stress levels. As expected, all tested Nrf2-pathway activators reduced the MitoSOX-calcein ratio (Fig. 3c), either to control levels, in the case of Ki 696–2, HPP-D and ezetimibe (p = 0.0056, p = 0.0038 and p < 0.0001 vs. annonacin, respectively), or to levels below control, in the case of licochalcone A (p < 0.0001 vs. control and p < 0.0001 vs. annonacin). Since increased oxidative stress is associated with oxidative DNA damage, we assessed the presence of 8-OHdG, a major marker of oxidative DNA lesion (Fig. 3d). We observed a significant 1.5-fold increase of 8-OHdG reactivity over control after annonacin exposure (p < 0.0001) (Fig. 3e). Surprisingly, the reactivity of 8-OHdG did not overlap with the nuclear DNA, stained with DAPI. This observation led us to speculate whether annonacin was mainly triggering mitochondrial oxidative DNA damage. To confirm this hypothesis, we co-targeted the cells against the outer mitochondrial membrane protein, TOM20. We observed a clear overlap between 8-OHdG and TOM20 reactivity, supporting our hypothesis that annonacin mainly induced mitochondrial oxidative DNA damage (Fig. 3d). In agreement with the reduction of mitochondrial oxidative stress observed upon treatment with Nrf2-pathway activators, treatment with Ki 696–2 and HPP-D promoted a small but significant reduction of 8-OHdG reactivity (p = 0.001, p = 0.044 vs. control and p = 0.017, p = 0.0004 vs. annonacin, respectively), whereas ezetimibe and licochalcone A had a more pronounced effect and contributed to levels of oxidative DNA damage comparable to healthy control cells (p = 0.300, p = 0.746 vs. control, respectively, and p < 0.0001 vs. annonacin for both compounds) (Fig. 3e). Overall, our data suggest that Nrf2-pathway activators lessened the mitochondrial oxidative burden and reduced the DNA damage induced by annonacin exposure.

### Assessing the effects of the Nrf2-pathway activators *on tau* splicing

**Fig. 4:**
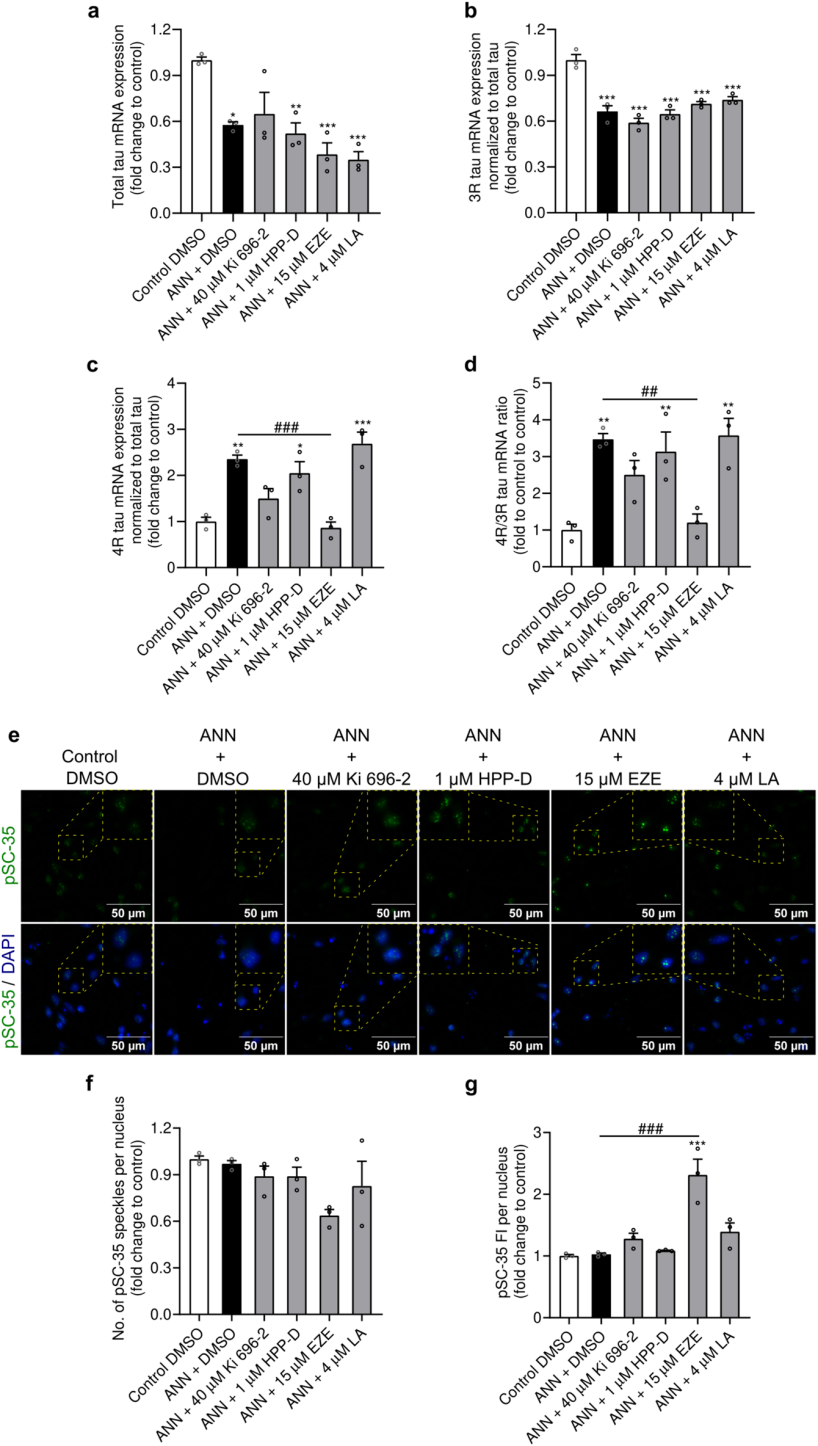
Effects of Nrf2-pathway activators in tau splicing. LUHMES cells were treated with protective concentrations of different Nrf2-pathway activators in the presence of 20 nM annonacin (ANN), in glucose-reduced medium for 48 h. mRNA expression of total Tau (**a**), and 3R (**b**) and 4R Tau isoforms (**c**) were determined by RT-qPCR. The ratio between 4 and 3R Tau isoforms is presented in (**d**) (ezetimibe: EZE; Licochalcone A: LA). Data are plotted as mean ± SEM (One-way ANOVA with Sidak’s post hoc test; N = 3). (**e**) Representative immunofluorescence images with 400 × magnification of pSC-35 antigen (green) with counterstained cell nuclei (blue) and respective merged channels are provided with a digital zoom (yellow dashes), for better visualization of pSC-35 speckles. Scale bar: 50 µm. Quantification of the number of pSC-35 speckles (**f**) and fluorescence intensity (FI) of pSC-35 (**g**) per nucleus, described in (**e**), expressed as fold change relative to control. Data are plotted as mean ± SEM (One-way ANOVA with Sidak’s post hoc test; N = 3). *p < 0.05, **p < 0.01 and ***p < 0.001 vs. control. ^##^*p* < 0.01 and ^###^*p* < 0.001 vs. annonacin.

Next, we investigated if the Nrf2-pathway activators had direct effects on the annonacin-induced changes of tau isoform expression. We started by determining the transcriptional levels of total tau, 3R- and 4R-tau isoforms. Levels of total tau transcripts were significantly downregulated in annonacin-only treated cells compared to control (p = 0.015) (Fig. 4a), likely resulting from the downregulation of 3R-tau transcripts induced by annonacin (p < 0.0001) (Fig. 4b), which were not influenced by any of the tested Nrf2-pathway activators. On the other hand, annonacin-exposure induced a significant upregulation of 4R-tau transcripts by twofold over control cells (p = 0.002) (Fig. 4c), consequently increasing the 4R-/3R-tau isoforms ratio to 3.5-fold over control (p = 0.003) (Fig. 4d). This finding mimicked the typical disruption of the equimolar ratio between 4R- and 3R-tau isoforms of a healthy adult human brain that occurs in 4R-tauopathies. Treatment with ezetimibe prevented the annonacin-induced upregulation of 4R-tau transcripts (p = 0.999 vs. control and p = 0.001 vs. annonacin) (Fig. 4c). As a consequence, not only was the 4R-/3R-tau isoforms ratio significantly lower in the ezetimibe-treated cells than in the annonacin-only treated cells (p = 0.006), but it also remained similar to healthy control cells (p > 0.999) (Fig. 4d). Considering that annonacin-induced 4R-tau upregulation was previously associated with the splicing factor SRSF2/SC-35, we wondered if the treatment with Nrf2-pathway activators, particularly with ezetimibe, would impact the expression or the nuclear distribution of SRSF2/SC-35 (Fig. 4e). Upon exposure to annonacin, we observed no changes in the number of pSC-35 speckles per nucleus (p > 0.999) (Fig. 4f), nor in its fluorescence intensity (p > 0.999) (Fig. 4g). Interestingly, treatment with ezetimibe led to a non-significant reduction of the number of speckles per nucleus (p = 0.053 vs. control and p = 0.086 vs. annonacin) (Fig. 4e), and a clear alteration of the nuclear distribution of pSC-35, promoting the formation of large and bright clusters, with a fluorescence intensity per nucleus twofold higher than control (p < 0.0001 vs. control and annonacin) (Fig. 4f). Collectively, our data suggest that ezetimibe modulated the expression of 4R-tau isoforms possibly through the splicing factor SRSF2/SC-35.

### Assessing the effects of the Nrf2-pathway activators *on tau* phosphorylation

**Fig. 5:**
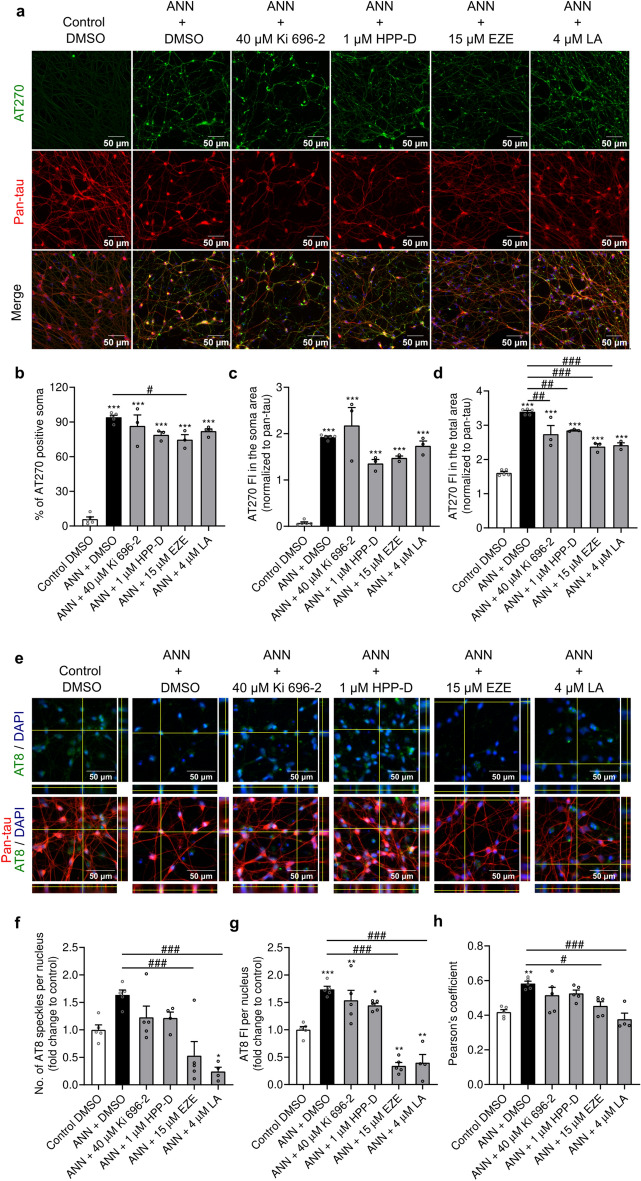
Effects of Nrf2-pathway activators in tau phosphorylation at epitope Thr-181 and at Ser-202/Thr-205. LUHMES cells were treated with protective concentrations of different Nrf2-pathway activators in the presence of 20 nM annonacin (ANN), in glucose-reduced medium for 48 h. (**a**) Representative immunofluorescence images with 200 × magnification of AT270 (pThr-181 tau) (green) and pan-tau antigen (red) with counterstained cell nuclei (blue) and respective merged channels are provided. Scale bar: 50 µm. Quantification of the number of AT270 positive somas (**b**) and fluorescence intensity (FI) of AT270 in the soma region (**c**) and total cell area (soma and neurites) (**d**) normalized to pan-tau (ezetimibe: EZE; Licochalcone A: LA). Data are plotted as mean ± SEM (One-way ANOVA with Sidak’s post hoc test; N = 3). (**e**) Representative orthogonal projections of immunofluorescence z-stack images with 400 × magnification of AT8 (pSer-202/Thr-205 tau) (green) and pan-tau antigen (red) with counterstained cell nuclei (blue) and respective merged channels are provided. Scale bar: 50 µm. Quantification of the number of AT8 speckles (**f**) and fluorescence intensity of AT8 (**g**) per nucleus, described in (**e**), expressed as fold change relative to control. Data are plotted as mean ± SEM (One-way ANOVA with Sidak’s post hoc test; N = 4). (**h**) Pearson´s coefficient values of AT8 with nuclei. Data are plotted as mean ± SEM (One-way ANOVA with Sidak’s post hoc test; N = 4). *p < 0.05, **p < 0.01 and ***p < 0.001 vs. control. ^#^*p* < 0.05, ^##^*p* < 0.01 and ^###^*p* < 0.001 vs. annonacin.

We also explored the impact of Nrf2-pathway activators on tau phosphorylation. Healthy control cells exhibited AT270 reactivity against pThr-181 tau almost exclusively in the neuritic compartment, with only 6% of the cellular somas being AT270-positive (Fig. 5a,b). Exposure to annonacin led to a significant accumulation of pThr-181 tau in the soma area, with more than 90% of the somas becoming AT270-positive (p < 0.0001). Consequently, AT270 reactivity normalized to pan-tau, in the soma area, significantly increased from a 0.07 ratio in control cells to a 1.9 ratio in annonacin-exposed cells (p < 0.0001) (Fig. 5c). Tau redistribution from the neuritic to the somatic compartment was modestly mitigated by ezetimibe (p = 0.016), which reduced the number of AT270-positive somas to 75% (Fig. 5b). When assessing the pThr-181 tau expression altogether, both in the neuritic and somatic compartment, we observed an increase in tau phosphorylation from a 1.6 ratio in control cells to a 3.4 ratio in annonacin-exposed cells (p < 0.0001) (Fig. 5d), when normalized to pan-tau reactivity. Treatment with the Nrf2-pathway activators attenuated annonacin-induced tau hyperphosphorylation (p = 0.001 for Ki 696-2, p = 0.006 for HPP-D, and p < 0.0001 for ezetimibe and licochalcone A vs. annonacin). Additionally, we also observed an increase in AT8 reactivity against pSer-202 and pThr-205 tau epitope, in the presence of annonacin (Fig. 5e). The AT8 reactivity was mainly observed as small speckles dispersed throughout the nuclear area. Exposure to annonacin had a non-significant propensity to increase the number of AT8 speckles per nucleus (p = 0.078 vs. control) (Fig. 5f), but contributed to a statistically higher AT8 fluorescence intensity (p = 0.0003 vs. control) (Fig. 5g). Treatment with Nrf2-pathway activators, particularly with ezetimibe and licochalcone A, led to a significant reduction of the number of AT8 speckles (p = 0.0005 for ezetimibe and p < 0.0001 for licochalcone A), as well as the AT8 fluorescence intensity (p < 0.0001 for both compounds) compared to annonacin. The effects of ezetimibe and licochalcone A were so pronounced that the AT8 reactivity in cells treated with these compounds was significantly lower than in control cells (p = 0.001 for ezetimibe and p = 0.005 for licochalcone A). Additionally, licochalcone A contributed to a significantly reduced number of AT8 speckles compared to the control condition (p = 0.035). We further confirmed our suspicions that annonacin was promoting nuclear expression of AT8 epitope by estimating the Pearson´s coefficient between AT8 reactivity and DAPI-stained nuclei, in cells exposed to annonacin. Indeed, we observed that annonacin-exposed cells exhibited a statistically higher Pearson´s coefficient than control cells (Pearson´s coefficient r = 0.42 in control condition vs. r = 0.58; p = 0.002) (Fig. 5h), thus suggesting that annonacin may have favored the nuclear expression of AT8 epitope. Interestingly, treatment with ezetimibe and licochalcone A contributed to reducing the Pearson´s coefficient values when compared with annonacin-only treated cells (r = 0.45, p = 0.980 vs. control and p = 0.022 vs. annonacin for ezetimibe; r = 0.38, p = 0.969 vs. control and p = 0.0003 vs. annonacin for licochalcone A). Taken altogether, our data suggests that treatment with Nrf2-pathway activators attenuated the annonacin-induced changes in tau phosphorylation and tau subcellular redistribution, in a compound-specific manner.

### Assessing the activation of the Nrf2 pathway

**Fig. 6:**
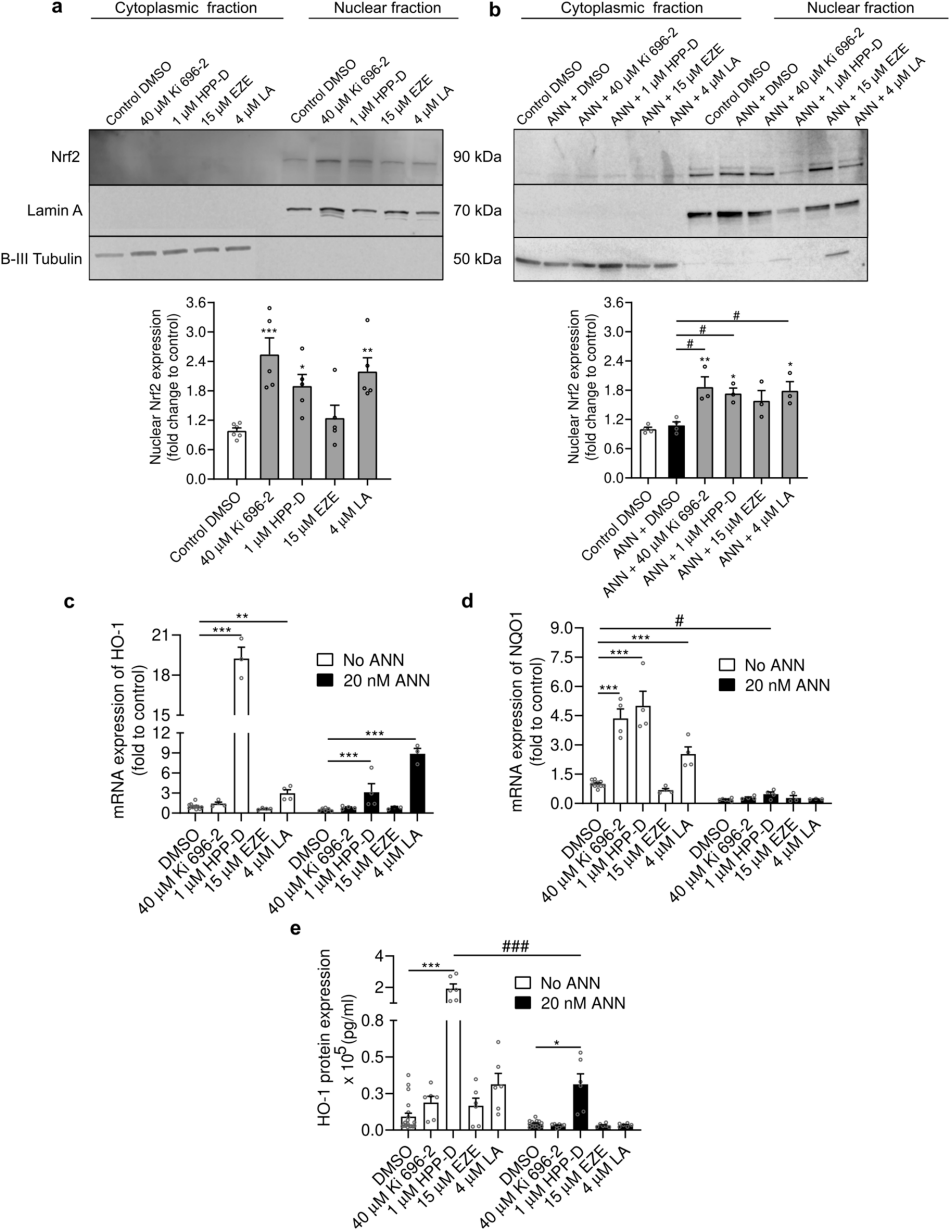
Treatment with Nrf2-pathway activators induced Nrf2 nuclear translocation and expression of Nrf2-target genes. LUHMES cells were treated with protective concentrations of different Nrf2-pathway activators in glucose-reduced medium for 4 h, in the absence (**a**) or presence (**b**) of 20 nM annonacin (ANN) (ezetimibe: EZE; Licochalcone A: LA). Cells were harvested and subjected to biochemical fractionation. Fractions were analyzed by 7.5% Tris–glycine SDS PAGE and Western blotting. A representative blot for each experimental condition is provided. The original uncropped blots from (**a**) and (**b**) are presented in Supplementary Figs. S2 and S3 online, respectively. Densitometric analysis of Western blots are plotted as mean ± SEM. (**a**) (One-way ANOVA with Dunnett’s post hoc test; N = 5). (**b**) (One-way ANOVA with Sidak’s post hoc test; at least N = 3). mRNA expression of the Nrf2-target genes *HO-1* (**c**) and *NQO1* (**d**) were analyzed by reverse transcription quantitative PCR (RT-qPCR) after 48 h treatment with protective concentrations of different Nrf2-pathway activators, in the absence or presence of 20 nM ANN, in glucose-reduced medium. The y-axis is presented discontinuously in panels (**c**) and (**e**). Data are plotted as mean ± SEM. (Two-way ANOVA with Sidak’s post hoc test; N = 3). Protein expression levels of HO-1 (**e**) were analyzed by ELISA after 48 h treatment in glucose-reduced medium with protective concentrations of different Nrf2-pathway activators, in the absence or presence of 20 nM annonacin. Data are plotted as mean ± SEM. (Two-way ANOVA with Sidak’s post hoc test; N = 3). *p < 0.05, **p < 0.01 and ***p < 0.001 vs. control. ^#^*p* < 0.05 vs. annonacin.

We then decided to confirm if the protectiveness of the Nrf2-pathway activators was associated with an activation of the Nrf2 pathway. For this purpose, we assessed the nuclear translocation of Nrf2 upon a 4 h treatment with the selected Nrf2-pathway activators, in the absence or presence of annonacin. The successful isolation of cytoplasmic and nuclear fractions was done qualitatively, by confirming the absence of a significant presence of lamin A (nuclear marker) in the cytoplasmic fraction, and absence of β-III tubulin (cytoplasmic marker) in the nuclear fraction. The Nrf2 protein was exclusively detected in the nuclear fraction as a single band at 90 kDa (Fig. 6a,b and Supplementary Fig. S2 and S3 online). The densiometric analysis of Nrf2 expression, normalized to lamin A, confirmed a significantly increased expression of Nrf2 protein in the nuclear fraction upon treatment with Ki 696–2 (p = 0.0006), HPP-D (p = 0.044) and licochalcone A (p = 0.006) (Fig. 6a), in the absence of annonacin. Similarly, in cells exposed to annonacin, Ki 696–2 (p = 0.0051 vs. control and p = 0.0180 vs. annonacin), HPP-D (p = 0.0187 vs. control and p = 0.0405 vs. annonacin) and licochalcone A (p = 0.0107 vs. control and p = 0.0231 vs. annonacin) also led to a higher expression of Nrf2 in the nuclear fraction, suggesting an increase in Nrf2 nuclear translocation (Fig. 6b). Treatment with ezetimibe showed no evidence of increasing the Nrf2 nuclear expression when compared with the control condition (p = 0.864), in the absence of annonacin, nor in the presence of annonacin (p = 0.0845 vs. control and p = 0.1752 vs. annonacin). To further confirm the activation of the Nrf2 pathway upon treatment with the selected Nrf2-pathway activators, we assessed the transcriptional expression of two Nrf2-positively regulated genes, *HO-1* and *NQO1*, both in the absence and presence of annonacin. The compound HPP-D led to an outstanding and very significant induction of *HO-1* expression (19-fold over DMSO control; p < 0.0001) and a milder increase in the transcription of *NQO1* (fivefold over DMSO control; p < 0.0001), in the absence of annonacin (Fig. 6c,d). Both compounds Ki 696–2 (p < 0.0001) and licochalcone A (p = 0.0002) significantly induced the transcription of *NQO1* in the absence of annonacin. This effect was more pronounced with Ki 696–2 (4.4-fold) than with licochalcone A (2.5-fold), when compared with DMSO control (Fig. 6d). In the presence of annonacin (DMSO control condition), we observed a downregulation of both target-genes (p = 0.855 for *HO-1* and p = 0.039 for *NQO1*) compared to DMSO control in the absence of annonacin (Fig. 6c,d). The expression level of *HO-1* was only significantly rescued from this downregulation by HPP-D (p = 0.0007) and licochalcone A (p < 0.0001) treatment, compared with DMSO control condition under annonacin intoxication (Fig. 6c). Treatment with ezetimibe did not induce significant changes in the expression of *HO-1* or *NQO1*, neither in the absence (p = 0.947 for *HO-1* and p = 0.811 for *NQO1*) nor presence (p = 0.993 for *HO-1* and p = 0.999 for *NQO1*) of annonacin, when compared with the respective DMSO control conditions (Fig. 6c,d).

The correlation between HO-1 transcriptional and translational levels was confirmed by ELISA. Only HPP-D significantly increased the HO-1 protein expression, both in the absence (p < 0.0001) and presence (p = 0.047) of annonacin (Fig. 6e). However, this upregulation was less pronounced under annonacin intoxication (p < 0.0001 when compared to HPP-D-treated cells in the absence of annonacin). To rule out the existence of a Nrf2-independent protective mechanism, we performed cell viability experiments in Nrf2-knockdown cells (Supplementary Fig. S4 online). Treatment with Nrf2 siRNA successfully downregulated the transcriptional levels of *Nrf2* (p = 0.0008 vs. NTC and negative control siRNA) and its gene-target *NQO1* (p = 0.0105 vs. NTC and p = 0.014 vs. negative control siRNA) (Supplementary Fig. S4a online), as well as the Nrf2 protein levels (p = 0.009 vs. NTC and p = 0.002 vs. negative control siRNA) (Supplementary Fig. S4b, S4c and S5 online). Only the protective effects of HPP-D and licochalcone A against annonacin toxicity were slightly attenuated in Nrf2-knockdown cells (Supplementary Fig. S4d online). We excluded a lack of efficiency of the siRNA treatment under the annonacin exposure by confirming a downregulation of the *Nrf2* transcription (Supplementary Fig. S4e online). However, we observed that despite the knockdown, treatment with Nrf2-pathway activators, such as licochalcone A, still led to an induction of *NQO1* expression (Supplementary Fig. S4f online). Overall, although our data demonstrated an activation of the Nrf2 pathway upon treatment with Ki 696–2, HPP-D and licochalcone A, the evidence was insufficient to conclude if the protectiveness of the Nrf2-pathway activators was exclusively associated with a direct activation of the Nrf2 pathway.

## Discussion

Compromised bioenergetic capacity and antioxidant defense are associated with increased oxidative stress and tau phosphorylation, consequently promoting neurodegeneration^1,24^. In brains from AD patients, the Nrf2 signaling pathway, a key-player of the antioxidant response and cellular metabolism, was found dysregulated and associated with exacerbated signs of neuroinflammation^7–10^. Evidence that activation of the Nrf2 pathway promotes resilience against oxidative stress, and reduces tau hyperphosphorylation and microglia activation, supports the idea of a neuroprotective role of Nrf2-pathway activators in tauopathies and possibly in other neurodegenerative proteinopathies^11–13^. While compounds derived from licochalcone A have been shown to reduce neuroinflammation^25^ and to attenuate beta-amyloid aggregation^26^, a central player of AD, evidence about the protectiveness of other Nrf2-pathway activators in tauopathies, such as Ki 696 or HPP-D, is very limited as almost no studies are currently available. For this reason, we explored the neuroprotective potential of mechanistically diverse Nrf2-pathway activators against annonacin toxicity, a mitochondrial complex I inhibitor known to induce common features of tau pathology, such as energetic impairment and changes in tau’s expression, phosphorylation and location^22,23,27,28^. Similar to previous reports, we observed altered tau splicing, increased tau phosphorylation and tau redistribution from the axonal to the somatic compartment, accompanied by neurite degeneration and neuronal death, in neurons exposed to annonacin. Additionally, annonacin triggered mitochondrial depolarization and impaired ATP production, ultimately promoting oxidative stress and inducing oxidative DNA damage. Treatment with the tested Nrf2-pathway activators, particularly the p62-activators (ezetimibe and licochalcone A) reduced the oxidative stress and attenuated the oxidative DNA damage triggered by annonacin insult. Moreover, the Nrf2-pathway activators partially prevented neurite degeneration and promoted cell viability. Interestingly, we observed a heterogeneous level of protection among the different compounds. For example, while p62-activators yielded the lowest rates of neuronal death after exposure to annonacin, Fyn kinase inhibitors, such as PP2 and saracatinib, showed no evidence of neuroprotection. We also observed that only the Bach1 inhibitor, HPP-D, was capable of ameliorating the annonacin-induced mitochondrial dysfunction by partially preventing mitochondrial depolarization and improving ATP production. Curiously, improved mitochondrial function did not correlate with higher cell viability. In fact, licochalcone A and ezetimibe, the two compounds that mostly avoided annonacin-induced neuronal death, were associated with lower MTT reduction and aggravated mitochondrial depolarization. These effects may be related to their known capacity to activate the AMPK (AMP-activated protein kinase) pathway, a sensor of metabolic and energy status^18,29^. Pharmacological activation of AMPK has been previously associated with mitochondrial depolarization and attenuated mitochondrial respiration^29^. It is reasonable to speculate that licochalcone A and ezetimibe may have contributed to further diminishing the mitochondrial performance via AMPK-activation. Overall, these results may indicate that mitochondrial rescue may not be a key-factor for neuroprotection against annonacin toxicity.

With the exception of ezetimibe, the protective Nrf2-pathway activators promoted Nrf2-nuclear translocation, regardless of the exposure to annonacin. The increase of Nrf2-nuclear expression was associated with an increase in the expression of Nrf2-positive regulated genes, mainly in the absence of annonacin. In fact, annonacin-exposure led to a downregulation of *NQO1* and *HO-1* mRNA, with the latter being rescued only by HPP-D and licochalcone A, possibly indicating that the initial Nrf2-activation was not sustainable at a later timepoint. This observation made us question the existence of Nrf2-independent protective mechanisms, particularly for ezetimibe. Supporting our hypothesis, we did not observe an abolishment of the neuroprotective effects of ezetimibe in Nrf2-knockdown cells. Indeed, only the protective effects of HPP-D and licochalcone A were marginally diminished in Nrf2-knockdown cells, suggesting that the protectiveness of the remaining compounds may be independent of, or only partially dependent on the Nrf2 activation. Due to an apparent insufficient Nrf2-knockdown, we were not capable to definitely exclude the existence of an alternative protection mechanism independent of Nrf2 activation. Further investigation in Nrf2-knockout neurons will be needed to clarify how much the protectiveness of the Nrf2-pathway activators is directly dependent on the Nrf2 activation. Surprisingly, we found that ezetimibe, an approved drug for the treatment of hypercholesterolemia, prevented the upregulation of 4R-tau isoform transcripts induced by annonacin, and kept the ratio of 4R-/3R-tau isoforms similar to healthy control cells. Although it should be noticed that an increase in 4R-tau expression is not strictly pathological, since it is also associated with neuronal maturation^30^, exposure to annonacin was found to upregulate 4R-tau isoforms via modulation of the splicing factor SRSF2/SC-35, which promotes inclusion of *MAPT* exon 10 and consequently increases 4R-tau transcripts^15,23^. Noteworthy, in brains from patients with PSP, the upregulation of 4R-tau expression was specifically associated with increased expression of SRSF2/SC-35^23^. Interestingly, ezetimibe was the only test compound that altered the nuclear distribution of SRSF2/SC-35. The formation of enlarged and brighter speckles, which we observed in the presence of ezetimibe, is known to occur when the transcription activity is halted by transcription or splicing inhibitors^31,32^. These dynamic structures are described as storage sites of splicing factors. Particularly, recent studies demonstrated that AMPK regulates the splicing activity of SRSF1 and SRSF9, both known for promoting 4R-tau transcripts^33,34^. Direct phosphorylation of SRSF1 and SRSF9 by AMPK inhibits their interaction with RNA, consequently suppressing their splicing activity. Based on our preliminary observation that ezetimibe, a known activator of AMPK^18^, prevents annonacin-induced upregulation of 4R-tau transcripts and promotes a phenotypic nuclear distribution of SRSF2/SC-35 typical of transcription and splicing arrest, we speculate that AMPK activation by ezetimibe may suppress the splicing activity of SRSF2/SC-35, consequently blocking the annonacin-induced upregulation of 4R-tau isoforms. The preliminary nature of this finding requires further investigation to confirm if SRSF2/SC-35 is a direct substrate of AMPK phosphorylation, and if the abolishment of annonacin-induced 4R-tau upregulation in the presence of ezetimibe is performed through modulation of SRSF2 activity.

Considering that annonacin exposure has been extensively associated with tau hyperphosphorylation and somatic accumulation^15,22,28^, we analyzed the effects of the Nrf2-pathway activators on tau phosphorylation at two particular epitopes, both considered as biomarkers of AD^35–37^. Specifically, we assessed the phosphorylation levels at the epitope Thr-181 (AT270), deemed to be a disease progression predictor in PSP^36^, and at the doubled-phosphorylated epitope Ser-202/Thr-205 (AT8), associated with aging and AD^37^. Interestingly, whereas all the protective Nrf2-pathway activators attenuated the annonacin-induced phosphorylation at epitope Thr-181 (AT270), only the p62-activators (ezetimibe and licochalcone A) reduced the phosphorylation at the epitope Ser-202/Thr-205 (AT8). Moreover, our data suggest that annonacin-exposure led to a nuclear accumulation of AT8 tau. Recently, nuclear accumulation of AT8 tau was reported in nutrient-deprived neurons and proposed to contribute to stabilizing the pro-apoptotic transcription factor p53, consequently promoting neurodegeneration^38^. Particularly, we observed that ezetimibe and licochalcone A appear to reduce nuclear accumulation of AT8. Currently, it is unknown if the AT8 nuclear accumulation represents a crucial event for the annonacin toxicity. Interestingly, frontotemporal dementia-*MAPT* mutations were also found to promote tau phosphorylation at the epitope Ser-202/Thr-205 (AT8) and tau nuclear mis-localization^39^, consequently leading to dysfunction of the nuclear membrane caused by altered microtubule dynamics. Further studies will be required to understand if AT8-positive tau interacts with the microtubules, and if its nuclear accumulation constitutes an important event of tau pathology, and perhaps a relevant therapeutic target. Nevertheless, considering the higher impact of ezetimibe and licochalcone A in preventing annonacin-induced neurodegeneration, we can speculate about the existence of a protective mechanism that is not restricted to the activation of the Nrf2-pathway. In fact, considering that ezetimibe and licochalcone A are known activators of AMPK, which in turn activates the Nrf2 pathway by promoting its release from KEAP1 repression, via p62 phosphorylation^18,40^, we speculate that the protective effects of these p62-activators may be related to AMPK-dependent apoptosis blockage. Indeed, activation of AMPK has been shown to inhibit cell death in nutrient-deprived cells^41^. However, it is noteworthy to mention that in our study we used immortalized human fetal cells. Fetal human brain cells almost exclusively express the shortest tau isoform (0N3R) and exhibit higher levels of phosphorylated tau than adult brain cells^38–41^. Therefore, it would be important to confirm our findings in a cell model with a tau expression and phosphorylation pattern more similar to the adult human brain.

In conclusion, our work suggests that Nrf2-pathway activators have a heterogeneous neuroprotective effect against annonacin, depending on their intrinsic mechanism of action. This observation may point to the existence of a tight regulation of the Nrf2-pathway, suggesting that Nrf2 activation has a differential outcome depending on the activation mechanism that is pharmacologically modulated. Overall, despite the tested Nrf2-pathway activators having shown a neuroprotective role against oxidative stress and tau phosphorylation triggered by annonacin, supporting a therapeutic potential of Nrf2-pathway activators in the context of tauopathies, our data suggest that these neuroprotective effects may not be exclusively related to the Nrf2 pathway. Particularly, in the case of the p62-activators, a simultaneous activation of the Nrf2 and the AMPK pathways might be responsible for their outstanding neuroprotection in the studied paradigm. Further research will be needed to fully understand the therapeutic potential of synergistic promotion of the metabolic adaptation and cell survival as a strategy to develop efficient therapies for tauopathies, and eventually other neurodegenerative diseases.

## Methods

### Cell culture

We used LUHMES (lund human mesencephalic) cells derived from female human embryonic ventral mesencephalic cells by conditional immortalization (Tet-off v-myc overexpression). Cells were grown in monolayer at 37 °C in a humidified atmosphere of 5% CO_2_, in 75 cm^2^ tissue culture flasks coated with 0.05 µg/ml PLO (poly-l-ornithine). Cells were maintained in growth medium of DMEM/F12 (Sigma-Aldrich, St. Louis, MO, USA), supplemented with 1% N-2 supplement (Gibco, Thermo Fisher Scientific, Waltham, MA, USA) and 0.04 µg/ml basic fibroblast growth factor (PreproTech EC, Waltham, MA, USA), and routinely dissociated with trypsin–EDTA solution and subcultured when reaching 70% confluence to avoid overgrowth. For the experiments, we seeded 150,000 cells/cm^2^ in multi-well plates, double-coated with 0.05 µg/ml PLO and 5 µg/ml FN (fibronectin). The cells were allowed to differentiate for 8 days in differentiation medium of DMEM/F12 supplemented with 1% N-2 supplement, 1 µg/ml tetracycline, 2 mg/ml recombinant human GDNF (R&D Systems, Inc., Minneapolis, MN, USA), and 490 µg/ml dbcAMP (Sigma-Aldrich) into post-mitotic neurons^42^ with a dopaminergic phenotype^43^. To ensure maintenance of the nutritional levels, half of the medium was removed and replaced with fresh differentiation medium four days after seeding.

### Annonacin titration

Commercial annonacin (Cayman Chemical, Michigan, MI, USA) was diluted in DMSO and stored in small aliquots of 100 µM stock solution at -80 °C. For annonacin titration, LUHMES cells were seeded onto a 48-well microplate and intoxicated on day 6 of differentiation with different concentrations of annonacin, over a period of 48 h, in glucose-reduced differentiation medium of DMEM without glucose (Gibco), supplemented with 2.4 mM glucose, 1% N-2 supplement, 1 µg/ml tetracycline, 2 mg/ml GDNF, and 490 µg/ml dbcAMP to mimic the physiological concentration of glucose in the human brain^44,45^. Each experimental condition was run in triplicates. Cell viability was measured by calcein viability assay, as detailed below. An appropriate concentration was chosen in order to assure approximately 50% cell viability under the annonacin condition.

### Calcein viability assay

The calcein AM green dye (Thermo Fisher Scientific) was diluted in DMSO to obtain a 1 mM stock solution. The stock solution was further diluted in HBSS (Hanks' balanced salt solution), immediately before incubation, to achieve a final working concentration of 1 μM in the well. The medium was removed, the cells were washed once with warm HBSS, and 200 µl of calcein working solution was added to each well. For background correction, 200 µl of calcein working solution was added to a well without cells (background control). After 30 min of incubation at 37 °C, the calcein solution was removed and the cells were washed once with HBSS. Cells were lysed in a 200 µl volume of 1% Triton™ X-100 in PBS (phosphate buffered saline) and the lysate transferred to a 96‐well black plate for the readout. The fluorescence signal was measured on a FLUOstar® Omega plate reader (BMG LabTech, Ortenberg, Germany) at λ Ex/Em = 485/530 nm and data was analyzed with the MARS data analysis software (BMG LabTech). For background correction, we subtracted the background control signal from the measured values of all experimental conditions. Data were expressed as percentage of viability relative to the control (DMSO-treated) condition.

### Pharmacological treatments

For pharmacological treatments, we acquired several previously described Nrf2-pathway activators: ezetimibe (Cayman), licochalcone A (MedChem Express, New Jersey, NJ, USA), bardoxolone methyl, saracatinib and PP2 (AbMole BioScience Inc., Houston, TX, USA). The enantiomers Ki 696–1 and Ki 696–2, and the compound HPP-D were kindly provided by Orion Pharma (Espoo, Finland). All compounds were dissolved in DMSO and stored in small aliquots at -80 °C to avoid freeze–thaw cycles. Compound general toxicity was determined on PLO/FN double-coated 48-well microplates as follows: after 4 days of differentiation, LUHMES cells were incubated with the different Nrf2-pathway activators at concentrations ranging from 1 nM to 100 µM, for 96 h. The cell viability was assessed by calcein assay on day 8 of differentiation. Non-toxic concentrations of the Nrf2-pathway activators were selected and used in the pharmacological treatments, in combination with 20 nM of annonacin, in glucose-reduced differentiation medium, over a period of 48 h, similar to an in vitro model reported before^15^. The protective effects of Nrf2-pathway activators against annonacin toxicity were determined in terms of cell viability with the calcein assay.

### Neurite arborization analysis

Cells were seeded in differentiation medium, onto 24-well microplates containing PLO/FN coated 13 mm Ø coverslips, and submitted to the described pharmacological treatments for 48 h. After this period, the medium was removed and cells were washed once with warm HBSS, and fixed with 4% PFA (paraformaldehyde) in PBS, for 20 min at RT (room temperature). After fixation, cells were washed twice with PBS and permeabilized with 0.1% Triton™ X-100 in PBS, for 20 min at RT. Next, cells were blocked in 1.5% NHS (normal horse serum) in PBS, for 1 h at RT, and incubated overnight with 1:1,000 Tuj-1 Alex 549-conjugated antibody (#801208, BioLegend, San Diego, CA, USA) in 1.5% NHS in PBS. After washing three times with PBS, cell nuclei were stained with 1:5,000 DAPI (4ʹ,6-diamidino-2-phenylindole, Thermo Fisher Scientific) in PBS, for 15 min at RT. The coverslips were mounted in mowiol, and left to dry for 24 h before imaging. Ten to fifteen images per condition, across triplicates, from different fields of view were taken on a fluorescence microscope Leica-DFC9000GTC (Leica) with 200 × magnification (20 × objective, 10 × intermediate magnification). Identical acquisition parameters were used for all images. Neurites density and arborization were quantified on TIFF exported images using the “neurite analyzer” plugin described before^46^. The average value of the different view fields per experimental condition was taken and reported as fold change compared to the control (DMSO-treated) condition.

### Determination of mitochondria membrane potential

In PLO/FN-coated 24-well microplates, cells were simultaneously incubated with 20 nM of annonacin and the selected concentrations of Nrf2-pathway activators, in glucose-reduced medium, over the course of 24 h. After that, the medium was removed and cells were washed once with warm HBSS, followed by incubation with 5 µM JC-1 solution in HBSS, for 20 min at 37 °C. For the positive control, the mitochondrial oxidative phosphorylation uncoupler, CCCP, was used at a concentration of 50 µM. After the incubation period, cells were washed with HBSS, and fresh HBSS was added to keep the cells viable during live-image acquisition. Image acquisition was done on a fluorescence microscope Leica-DFC9000GTC (Leica, Frankfurt, Germany). Environmental conditions were kept steady at 37 °C with a 5% CO_2_ atmosphere during the entire acquisition time. Ten to fifteen images from different fields of view, dispersed across triplicates, were acquired with 200 × magnification (20 × objective, 10 × intermediate magnification), using the same image acquisition parameters for all conditions. The fluorescence of JC-1 monomers was captured on the green channel (λ Ex/Em = 475/519 nm) and the fluorescence of JC-1 aggregates was capture on the red channel (λ Ex/Em = 555/594 nm). Quantification of FI (fluorescence intensities) of JC-1 monomeric and aggregated forms was done using the ImageJ software (http://rsb.info.nih.gov/ij/, ImageJ bundled with 64-bit Java 8). Briefly, TIFF exported images were submitted to background subtraction using the rolling ball method with a radius of 50 pixels. Channel-specific thresholds were applied to all images to define the ROI (region of interest) and FI were measured as integrated density. Data were reported as the ratio between JC-1 aggregates and monomers, obtained by dividing the FI of the JC-1 aggregated form by the FI of the monomeric form.

### Assessing MTT reduction and ATP production

Mitochondrial activity was estimated by the reduction of MTT (3-(4,5-dimethylthiazol-2-yl)-2,5-diphenyltetrazolium, Sigma-Aldrich). Briefly, LUHMES cells were seeded on PLO/FN-coated 48-well microplates, in triplicates. After a 24 h treatment with 20 nM of annonacin and the protective concentrations of Nrf2-pathway activators in glucose-reduced medium, cells were incubated with 0.5 mg/ml of MTT dissolved in PBS, at 37 °C for 30 min. After that, the medium was removed and the plate was frozen at -80 °C for 1 h, for cell lysing. Next, the plate was thawed and the MTT crystals were completely dissolved in 250 µl of DMSO with vigorous shaking. The absorbance was measured on a FLUOstar® Omega plate reader at λ = 590 nm, using λ = 630 nm absorbance as a background correction. The obtained results were analyzed with the MARS data analysis software (BMG LabTech). After background subtraction, the data were expressed as a percentage of MTT reduction relative to the control (DMSO-treated) condition. Determination of cellular ATP levels was done by using the ViaLight™ plus kit (Lonza, Basel, Switzerland), following the manufacturer’s instructions. Cells were seeded on PLO/FN-coated 48-well microplates and the pharmacological treatments were done similarly as to the assessment of the MTT reduction. On the next day, the medium was removed and cells were washed once with warm HBSS and rested in 210 µl HBSS. The plate was left to reach RT before adding 70 µl cell lysis reagent to each well, provided by the kit. After a 10 min incubation, 10 µl of each lysed cell extract was transferred to a 384-well white plate and combined with 10 µl of ATP monitoring reagent plus. The plate was incubated in the dark for 10 min at RT before the luminescence signal was measured on a FLUOstar® Omega plate reader. The obtained results were analyzed with the MARS data analysis software (BMG LabTech) and expressed as RLUs. For background correction, the RLUs detected for the background control (i.e., 10 µl of ATP monitoring reagent plus combined with 10 µl of cell lysis reagent) were subtracted from the measured RLUs values of all testing conditions.

### Assessing mitochondrial reactive oxygen species

As before, cells seeded on PLO/FN-coated 24-well microplates were treated for 48 h with 20 nM of annonacin and the selected concentrations of Nrf2-pathway activators, in glucose-reduced medium. After this period, the medium was removed, and the cells were washed once with warm HBSS before being simultaneously incubated with 0.5 µM of MitoSOX™ red (Thermo Fisher Scientific) and 1 µM of calcein AM for 30 min at 37 °C. Next, the cells were again washed with HBSS and fresh HBSS was added to maintain cell viability during live-image acquisition on a fluorescence microscope Leica-DFC9000GTC (Leica). The cells were kept under controlled environmental conditions, and similar image acquisition parameters were used for all images, as explained before. We acquired ten to fifteen images per condition, from different view fields across triplicates, with a 200 × magnification (20 × objective, 10 × intermediate magnification). Quantification of MitoSOX and calcein AM FI was performed using the ImageJ software. Briefly, TIFF exported images were submitted to background subtraction using the rolling ball method with a radius of 50 pixels. Channel-specific thresholds were applied to all images to define the ROI, and the FI were measured as integrated density and reported as arbitrary units (a. u.). The MitoSOX FI was normalized to cell viability estimated by calcein AM FI, to obtain a MitoSOX FI/calcein FI ratio. The average ratio of the different view fields per experimental condition was taken and reported as fold change relative to the control (DMSO-treated) condition.

### Immunofluorescence

For immunostaining, cells were seeded in differentiation medium, on 24-well microplates containing PLO/FN-coated 13 mm Ø coverslips, and treated for 48 h as previously described. On the readout day, the medium was removed and cells were fixed, permeabilized and blocked as before. Primary antibody dilutions were made in 1.5% NHS in PBS (Supplementary Table S1) and incubated overnight at 4 °C. On the following day, the coverslips were washed three times with PBS and incubated with host-specific fluorescence secondary antibodies (Supplementary Table S1), for 2 h at RT, protected from light. After another three washes with PBS, cell nuclei were stained with 1:5,000 DAPI (4’,6-diamidino-2-phenylindole, Thermo Fisher Scientific) in PBS, for 15 min at RT. The coverslips were mounted in mowiol and left to dry for 24 h before imaging. Image acquisition for FI quantification was done with a 200 × magnification (20 × objective, 10 × intermediate magnification), or 400x (40 × objective, 10 × intermediate magnification) in the case of pSC-35 and AT8 immunostaining, on a fluorescence microscope Leica-DFC9000GTC (Leica). In the case of phospho-tau AT8, Z-stacks consisting of 18 plane images with a step size of 1 μm were acquired. The image acquisition parameters were identical for all conditions. Quantification of 8-OHdG (8-hydroxyguanosine) FI and tau species was performed using the ImageJ software. Ten-to-fifteen images per condition across triplicates, from different view fields, were used for this purpose. Briefly, TIFF exported images were submitted to background subtraction using the rolling ball method with a radius of 50 pixels. For 8-OHdG analysis, a channel-specific threshold was applied to all images to define the ROI and FI was measured and normalized to the cell number, determined by the DAPI channel. The average value of the different view fields per experimental condition was taken and reported as fold change compared to the control (DMSO-treated) condition. For the analysis of phospho-tau AT270 and pan-tau reactivity, channel specific thresholds were applied to all images. The DAPI channel was used to define nuclei from viable cells, by selecting particles with a size above 15 µm^2^ and a circularity of 0.3–1.0. The obtained mask was submitted to “close” and “fill holes” processes to smooth the objects and fill in small holes, followed by nine “dilations” and one “watershed” to obtain a ROI covering exclusively the soma of viable cells. The obtained mask was combined with threshold images of phospho-tau AT270 and pan-tau in order to define the respective ROI of each tau species. These masks were used to quantify the number of somas expressing each of these tau species, as well as the respective FI. The number of AT270-positive somas was divided by the number of pan-tau-positive somas and expressed as a percentage. The AT270 FI was normalized to the pan-tau FI and the ratio presented as fold change relative to the control (DMSO-treated) condition. For quantification of pSC-35 and AT8 speckle number and FI, we used CellProfiler™ 4 (https://cellprofiler.org/, Broad Institute Inc., Cambridge, MA, USA) speckle counting pipeline^47^. Twenty-to-thirty images per condition across triplicates were used for this purpose. ImageJ BIOP JACoP plugin was used for estimating co-localization of AT8 with DAPI-stained nuclei. Data were reported as fold change compared to the control (DMSO-treated) condition, or as Pearsonʹs coefficient values in case the of AT8 co-localization data.

### Cell fractionation

For fractionation experiments, LUHMES were seeded on double-coated T25 flasks and submitted to pharmacological treatments. Afterwards, the medium was removed and cells were briefly washed with warm HBSS, and trypsinized for 5 min at 37 °C. The cells were pelleted at 500×*g* for 5 min at 4 °C, washed once with ice-cold PBS (phosphate buffered saline), and centrifuged at 500 g for 2 min at 4 °C. Nuclear protein extraction was achieved using the NE-PER™ nuclear and cytoplasmic extraction kit (Thermo Fisher Scientific), according to the manufacturer’s instructions. Briefly, the pellet was resuspended in 50 µl of CER I buffer, supplemented with Halt™ protease and phosphatase inhibitor cocktail (Thermo Fisher Scientific), and incubated on ice for 10 min, followed by the addition of 2.75 µl of CER II, and a 2 min incubation on ice after vortexing. The samples were then centrifuged at maximum speed for 10 min at 4 °C and the cytoplasmic fraction was saved. The nuclear pellet was washed with ice-cold PBS and resuspended in 25 µl of NER buffer supplemented with protease and phosphatase inhibitors, followed by 6 cycles of 10 min incubation on ice, with 15 s of vortexing between each cycle. Finally, the samples were centrifuged at maximum speed for 10 min at 4 °C and the nuclear fraction was transferred to a new tube. All protein extracts were stored long-term at − 80 °C.

### Protein quantification and Western-blot

The concentrations of protein extracts were determined by BCA (bicinchoninic acid) assay, using the Pierce™ BCA protein assay kit (Thermo Fisher Scientific), according to the manufacturer’s instructions. Absorbance values were measured on a FLUOstar® Omega plate reader. For Western blot analysis, sample volumes were diluted in PBS to ensure that equal amounts of total protein were loaded. For fractionated samples, 30 µg of cytoplasmic fraction and 15 µg of nuclear fraction were applied. Samples were denatured by heating at 95 °C for 5 min on a ThermoMixer C (Eppendorf, Wesseling-Berzdorf, Germany) with 4 × Laemmli sample buffer supplemented with 10% β-ME (β-mercaptoethanol). SDS–PAGE was performed using 7.5% Criterion™ TGX™ precast midi gels in a Tris–glycine-SDS running buffer (Bio-Rad). The proteins were blotted onto PVDF membranes, using a trans-blot turbo transfer system (Bio-Rad) at 25 V for 30 min. The membranes were blocked for 1 h at RT, with 3 × Roti®-Block solution (Carl Roth, Karlsruhe, Germany) or 5% nonfat-dry milk in 0.05% TBST (Tris-buffered saline with 0.05% Tween20), in the case of the Nrf2 antibody. Membranes were incubated at 4 °C overnight under gentle shaking with the appropriate primary antibody solutions as listed in Supplementary Table S2. On the following day, the membranes were washed with TBST, and incubated with the HRP-secondary antibodies for 2 h at RT (Supplementary Table S2). After further washing, the membranes were exposed to Clarity Western ECL Substrate (Bio-Rad), or SuperSignal West Femto Maximum Sensitivity Substrate (Thermo Fisher Scientific) in the case of the Nrf2 antibody. Chemiluminescence signal was detected with an Odyssey® Fc imaging system (LI-COR Biosciences, Bad Homburg, Germany) and analyzed by Image Studio™ software (LI-COR Biosciences). Fraction purity was confirmed and signal intensity normalized to housekeeping proteins, using β-III tubulin for the cytoplasmic fraction and lamin A for the nuclear fraction. Protein expression was expressed as fold change relative to the control (DMSO-treated) condition.

### mRNA extraction and quantitative RT-PCR

LUHMES cells were seeded on PLO/FN coated 6-well microplates and the pharmacological treatments were performed as previously explained. The cells were washed in HBSS, prior to being collected for mRNA isolation (RNeasy® Plus Mini Kit, Qiagen, Venlo, Netherlands), following the manufacturer’s instructions. The mRNA concentrations were determined on a Nano-Drop 2000c (Thermo Fisher Scientific) and 1 µg of mRNA was reverse-transcribed into cDNA with the iScript™ Reverse Transcription Supermix for RT-qPCR Kit (Bio-Rad) on a thermal cycler (SensoQuest, Göttingen, Germany). Real-time PCR was performed using SYBR™ Select Master Mix for CFX (Thermo Fisher Scientific) with 30 ng cDNA and 0.4 µM of primers against the respective target genes (Supplementary Table S3), in a 20 µl total volume, on a CFX96 Touch™ Real-Time Cycler (Bio-Rad). The relative expression of mRNA was calculated using the CFX Manager software (ΔΔCt method) from Bio-Rad, and the Ct values of the target-genes were normalized to the reference genes *RPL22* and *UBQLN1*. The data were reported as fold change relative to the control (DMSO-treated) condition.

### Quantification of HO-1 protein levels by ELISA

Protein expression levels of HO-1 were determined by ELISA (enzyme-linked immunosorbent assay) using a commercial kit (#ab207621, Abcam) according to the manufacturer's instructions. For this purpose, cells were seeded onto PLO/FN coated 6-well microplates. After the pharmacological treatments, cells were washed with warm HBSS, trypsinized and pelleted as before, and finally lysed in 100 µl cell extraction buffer PTR provided by the kit. Sample concentration was measured by BCA, as previously mentioned, and 1 µg of total protein extract was loaded into the respective wells. All samples were run in duplicates and the absorbances were measured on a FLUOstar® Omega plate reader at λ 450 nm. For data analysis, we used the MARS software (BMG LabTech). First, we calculated the means of the absorbances for each condition. The mean value of the blank control (i.e., a condition with only sample diluent buffer) was subtracted from the mean values of all standard and experimental conditions. A 4PL-curve fit was plotted on Prism 9 software (GraphPad Software, San Diego, CA, USA), using the blank-corrected mean values of standard conditions. The obtained curve was used to determine the HO-1 protein concentration, followed by multiplication by the dilution factor to obtain the concentration of the target-protein in each sample.

### Nrf2 knockdown by siRNA transfection

LUHMES cells seeded on PLO/FN coated 48-well microplates were left to differentiate for 5 days, before being transfected with 25 nM of NRF2-targeting (gene ID 4780) or negative control siRNA (siTOOLs Biotech, Planegg, Germany), in Opti-MEM + GlutaMax medium (Thermo Fisher Scientific) with 0.4% of lipofectamine RNAiMax. After 24 h, the medium was removed and replaced with glucose-reduced medium for the pharmacological treatments, similar as described in the previous sections. Cell viability was determined by calcein assay, after 48 h of treatment, as explained before.

### Statistical analysis

Graphical representation and statistical analysis were carried out with Prism 9 software (GraphPad). All groups were checked for normal distribution using the D’Agostino & Pearson method. Multiple comparisons between normally distributed data sets were done by one-way ANOVA or two-way ANOVA with Dunnett’s, Sidak’s or Tukey’s post hoc test. Groups without normal distribution were analyzed by non-parametric Kruskal–Wallis test or Brown-Forsythe and Welch ANOVA test with Dunnett´s T3 post hoc. The alpha value for all statistical tests was 0.05. A p-value < 0.05 was considered statistically significant. The specific statistical test used to generate each p-value is given in the respective figure caption.

### Supplementary Information

Supplementary Information.

## Data Availability

The authors confirm that all data underlying the findings described in this paper are fully available without restriction within the paper and its Supporting information files, and from the corresponding author upon reasonable request.
