# Supplementary material for: Exploring the neuroprotective potential of Nrf2-pathway activators against annonacin toxicity
Source: Sci Rep. 2024 Aug 29;14:20123. doi: 10.1038/s41598-024-70837-1 (PMC11362529; doi:10.1038/s41598-024-70837-1)
Supplement: Supplementary file 1 — Supplementary Information. [file 41598_2024_70837_MOESM1_ESM.docx]

# Exploring the neuroprotective potential of Nrf2-pathway activators against annonacin toxicity

# Márcia F.D. Costa^1,2,5^, Thomas W. Rösler^1,+^, Günter U. Höglinger^1,2,3,4,*,+^

^1^Department of Translational Neurodegeneration, German Center for Neurodegenerative Diseases, Munich, Germany

^2^Department of Neurology, School of Medicine, Technical University of Munich, Munich, Germany

^3^Department of Neurology, Hannover Medical School, Hannover, Germany

^4^Department of Neurology, Ludwig-Maximilians University, Munich, Germany

^5^Laboratory of Pharmacology, Department of Diagnostics and Public Health, University of Verona, Verona, Italy

*Corresponding author E-mail: Guenter.Hoeglinger@med.uni-muenchen.de

^+^ These authors contributed equally to this work

**Supplementary figures**

**
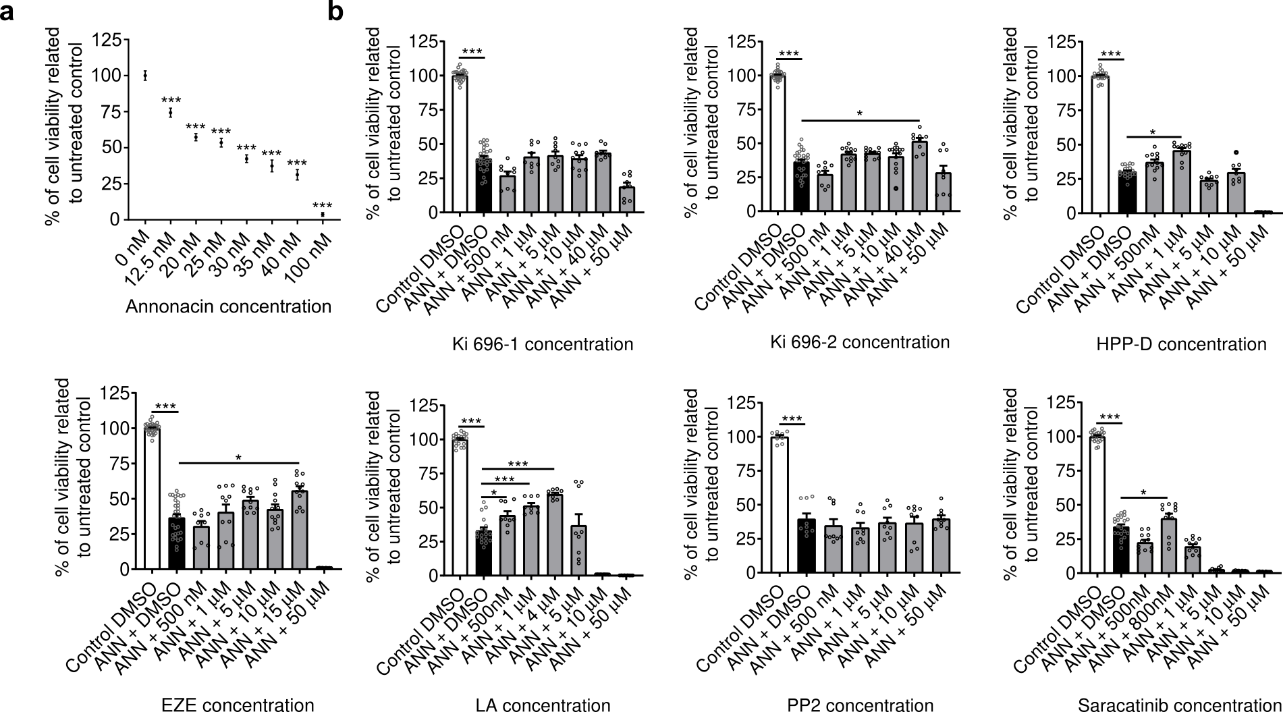
**

**Supplementary Fig S1. Annonacin titration and dose-response of the Nrf2-pathway activators.** (**a**) LUHMES cells were intoxicated with different concentrations of annonacin (ANN) in glucose-reduced medium for 48 h. Cell viability was measured by calcein fluorescence and reported as percentage of untreated control (0 nM). Data are plotted as mean ± SEM. ***p < 0.001 (One-way ANOVA with Dunnett’s post hoc test; N = 4). (**b**) Effects of different Nrf2-pathway activators on viability of cells exposed to annonacin. LUHMES cells were treated with different concentrations of each Nrf2-pathway activator in presence of 20 nM annonacin, in glucose-reduced medium for 48 h. Cell viability was measured by calcein fluorescence and reported as percentage of untreated control (ezetimibe: EZE; Licochalcone A: LA). Data are plotted as mean ± SEM. For visual simplification, statistical significances of non-protective compound concentrations are not reported. *p < 0.05, **p < 0.01 and ***p < 0.001 (One-way ANOVA with Dunnett’s post hoc test, except for the non-normal distributed datasets of Ki 696-2, HPP-D and ezetimibe, which were analyzed with non-parametric Kruskal-Wallis’s test; N = 3).


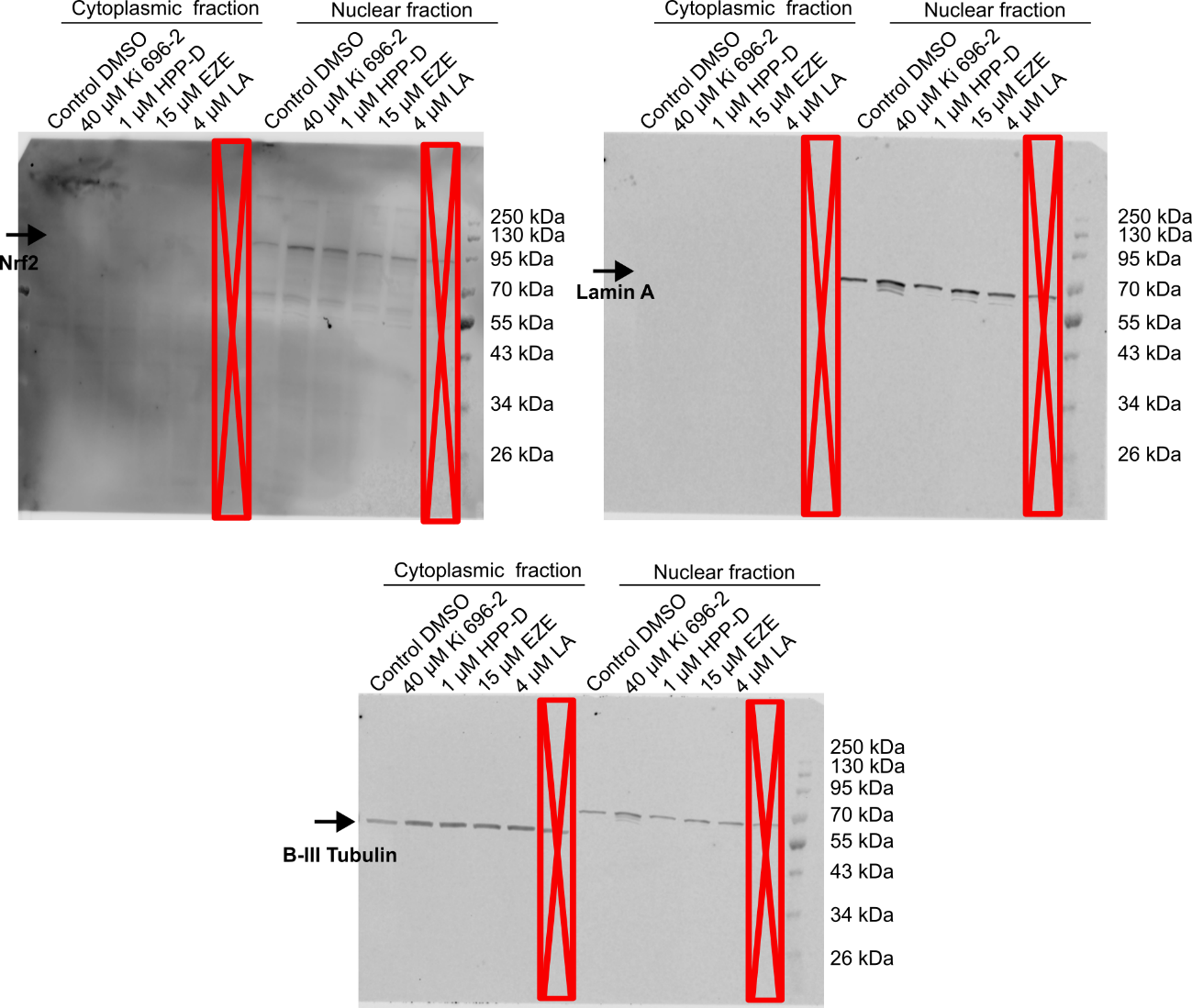


**Supplementary Figure S2. Immunoblots of fractionated cell lysates of LUHMES cells treated with Nrf2-pathway activators in the absence of annonacin.** The uncropped images of the immunoblot data displayed in Fig. 6a are here presented. Red marked lanes did not take part of the dataset (ezetimibe: EZE; Licochalcone A: LA).


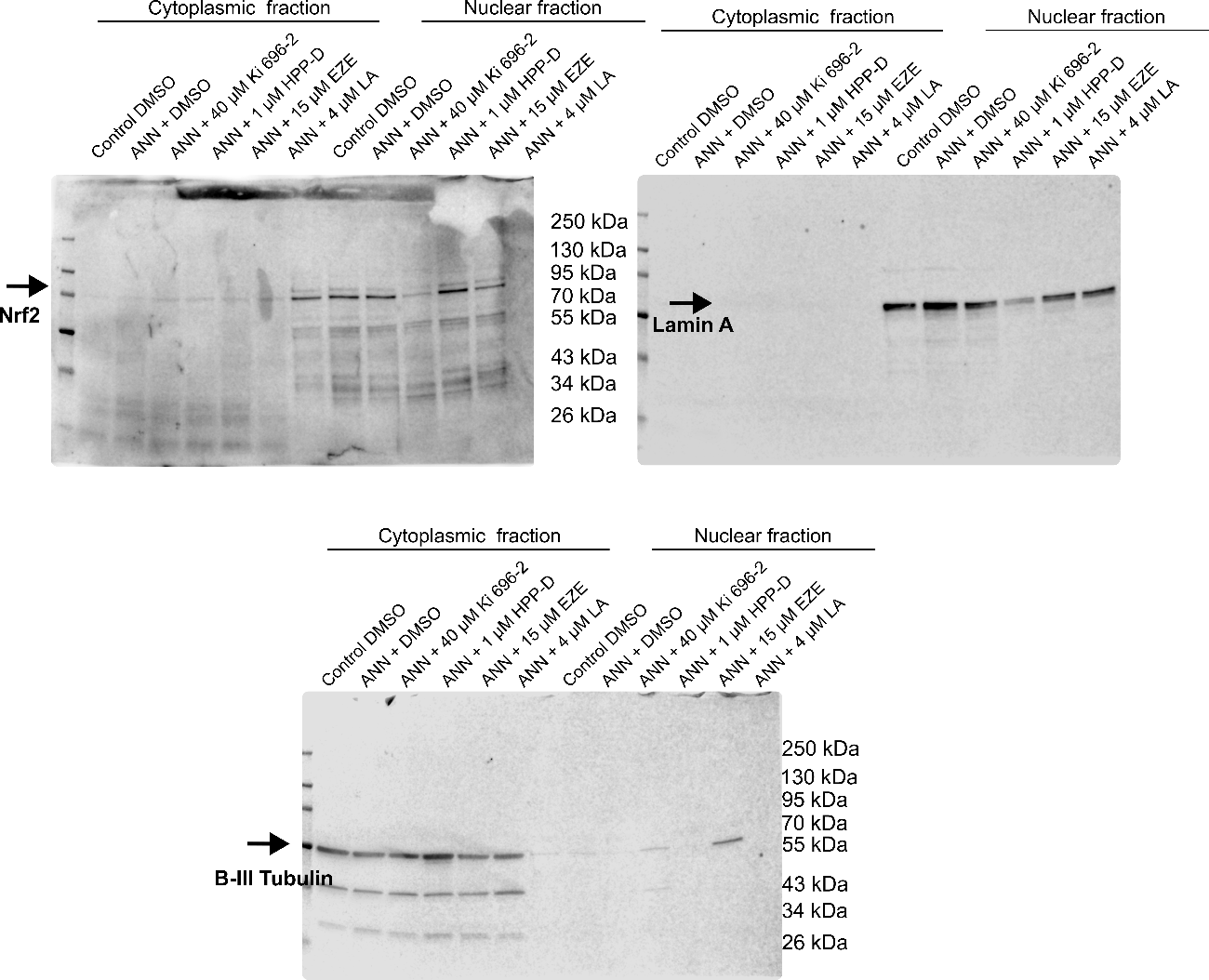


**Supplementary Figure S3. Immunoblots of fractionated cell lysates of LUHMES cells treated with Nrf2-pathway activators in the presence of annonacin.** The uncropped images of the immunoblot data displayed in Fig. 6b are here presented. (ezetimibe: EZE; Licochalcone A: LA).


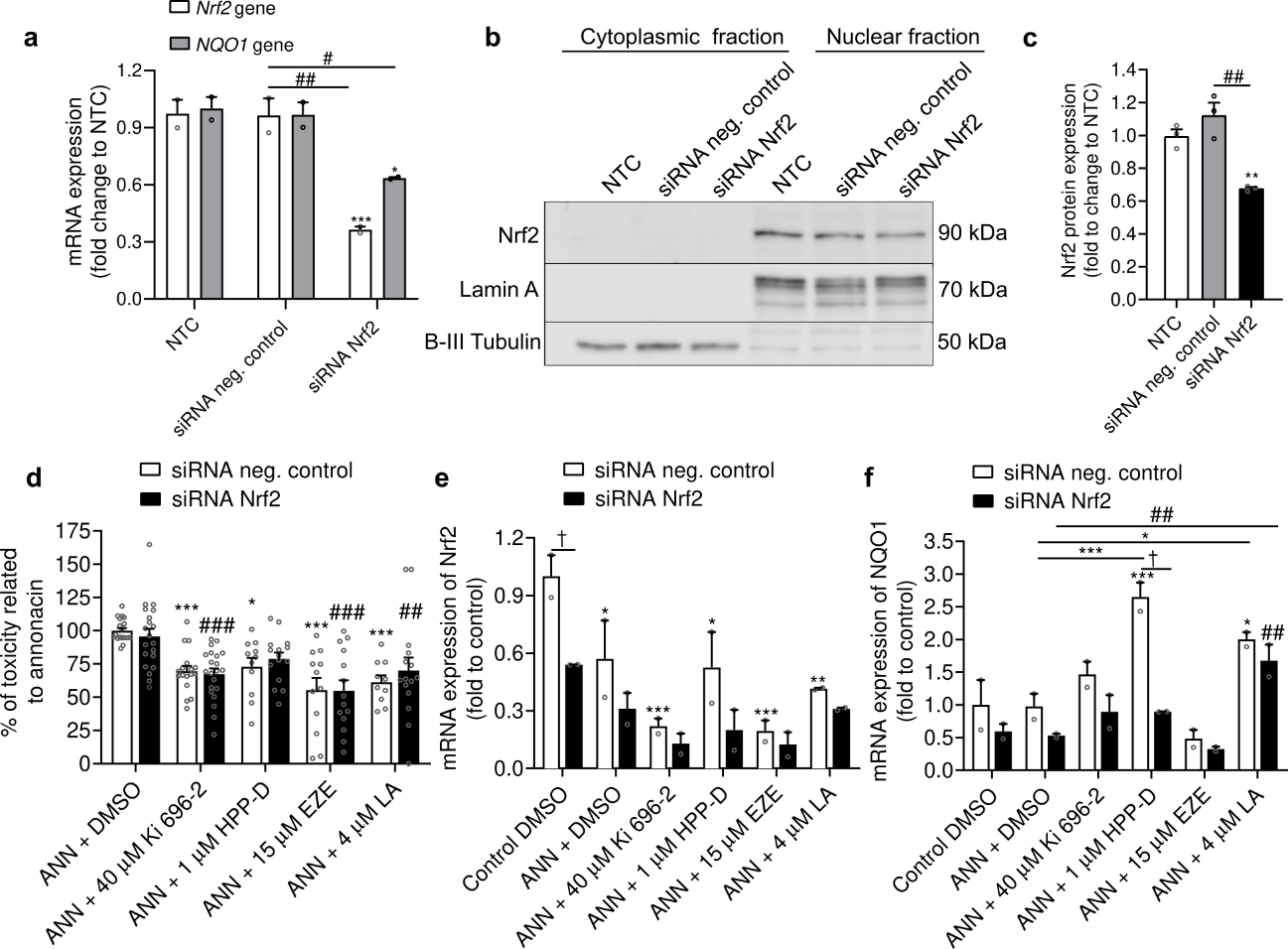


**Supplementary Fig S4. Nrf2 knockdown reduce the protectiveness of HPP-D and licochalcone A against annonacin toxicity.** LUHMEs cells were transfected with Nrf2 or negative control siRNA and harvested 48 h after transfection. (**a**) mRNA expression of Nrf2 and Nrf2-target gene *NQO1* were analyzed by RT-qPCR. Data are plotted as mean ± SEM. *p < 0.05, and ***p < 0.001 vs. non-transfected control (NTC) of the respective gene. #p < 0.05 and ##p < 0.01 (two-way ANOVA with Tukey´s post hoc test; N = 2). (**b**) Cytoplasmic and nuclear fractions were analyzed by 7.5% Tris-glycine SDS-PAGE and Western blotting. Blots are representative of 3 independent experiments. The original uncropped blot is presented in Supplementary Figure S5. (**c**) Densitometric analysis of Western blots described in (**b**). Data are plotted as mean ± SEM. **p < 0.01 vs. NTC, ##p < 0.01 (one-way ANOVA with Dunnett’s post hoc test; N = 3). (**d**) Nrf2-knockdown cells, and cells transfected with negative control siRNA were treated for 48 h with the selected Nrf2-pathway activators in presence of 20 nM annonacin (ANN), in glucose-reduced medium. Cell viability was measured by calcein fluorescence and expressed as percentage of annonacin toxicity (ezetimibe: EZE; Licochalcone A: LA). Data are plotted as mean ± SEM. **p < 0.01 and ***p < 0.001 vs. annonacin of negative control siRNA transfected cells. #p < 0.05, ##p < 0.01 and ###p < 0.001 vs. annonacin of Nrf2 siRNA transfected cells (two-way ANOVA with Dunnett’s post hoc test; N = 7). mRNA expression of Nrf2 (**e**) and NQO1 (**f**) were analyzed to confirm successful NRF2-knockdown in the annonacin model, described in (**d**). Data are plotted as mean ± SEM. *p < 0.05, **p < 0.01 and ***p < 0.001 vs. control of negative control siRNA transfected cells. ##p < 0.01 vs. control of Nrf2 siRNA transfected cells. †p < 0.05 (Two-way ANOVA with Sidak’s post hoc test for intragroup comparison (siRNA neg. control or siRNA Nrf2) with control condition and intergroup comparison (siRNA neg. control vs. siRNA Nrf2) between similar conditions, and Dunnett’s post hoc test for intragroup comparison with annonacin condition; N = 2).


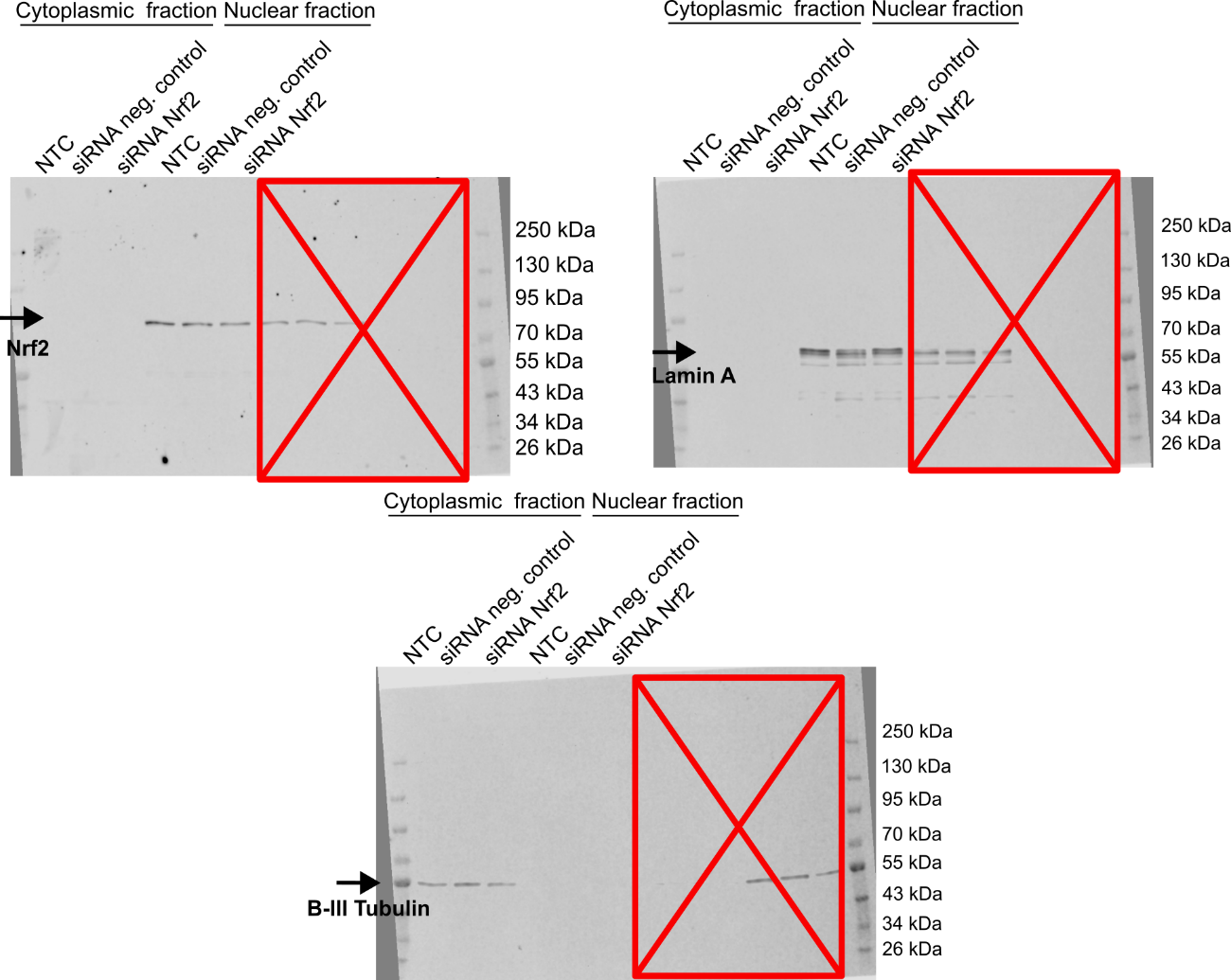


**Supplementary Figure S5. Immunoblots of fractionated cell lysates of LUHMES cells treated with Nrf2-targeted siRNA or respective negative control.** The uncropped images of the immunoblot data displayed in Supplementary Fig. S4b are here presented. Red marked lanes did not take part of the dataset. (Non-transfected control: NTC).

**Supplementary tables**

**Supplementary Table S1. List of antibodies used for immunocytochemistry.**

| **Primary antibody** | | | | |
| --- | --- | --- | --- | --- |
| **Antigen** | **Supplier** | **Catalog no.** | **Dilution** | **Solvent** |
| 8-OHdG | Santa Cruz (Dallas, USA) | sc-66036 | 1:250 | 1.5% NHS in PBS |
| TOM 20 (D8T4N) | Cell signaling | 42406 | 1:200 |  |
| pSC-35 | Santa Cruz | sc-53518 | 1:250 |  |
| AT270 (Tau pThr181) | Invitrogen | MN1050 | 1:400 |  |
| AT8 (Tau pSer-202 and pThr-205) | Invitrogen | MN1020 | 1:500 |  |
| Dako-Tau | Agilient | A0024 | 1:1,000 |  |
| **Secondary antibody** | | | | |
| AlexaFluor488  Mouse IgG | Thermo Fisher | A-21202 | 1:1,000 | 1.5% NHS in PBS |
| AlexaFluor594  rabbit IgG |  | A-21207 |  |  |
| AlexaFluor635  rabbit IgG |  | A-31577 |  |  |

**Supplementary Table S2. List of antibodies used for Western-blot.**

| **Primary antibody** | | | | |
| --- | --- | --- | --- | --- |
| **Antigen** | **Supplier** | **Catalog no.** | **Dilution** | **Solvent** |
| NRF2 (D1Z9C) | Cell signaling (Danvers, USA) | 12721 | 1:1,000 | 5% nonfat milk in 0.05% TBST |
| Dako-Tau | Agilient | A0024 | 1:10,000 | 5% BSA in 0.05% TBST |
| Lamin A (133A2) | Cell signaling | 86846 | 1:1,000 |  |
| Β-III tubulin (TU-20) | Merk | MAB1637 | 1:10,000 |  |
| **Secondary antibody** | | | | |
| Mouse IgG-HRP | Vector Laboratories, Burlingame, CA, USA | PI-2000-1 | 1:2,500 | 5% BSA in 0.05% TBST |
| Rabbit IgG-HRP |  | PI-1000-1 | 1:5,000 |  |

**Supplementary Table S3. List of primers used for quantitative RT-PCR.**

| **Target-gene** | **Forward primer (5´-3´)** | **Reverse primer (5´-3´)** |
| --- | --- | --- |
| *HO-1* | CAGGCAGAGAATGCTGAGTTC | AAGACTGGGCTCTCCTTGTTGC |
| *NQO1* | CCTGCCATTCTGAAAGGCTGGT | GTGGTGATGGAAAGCACTGCCT |
| *Nrf2* | TTCCCGGTCACATGAGAG | TCCTGTTTGCATACCGTCTAAATC |
| *Total MAPT* | AGAGTCCAGTCGAAGATTGGGTC | GGGTTTCAATCTTTTTATTTCCTCC |
| *3R MAPT* | *GAAGAATGTCAAGTCCAAGATCGG* | GACTATTTGCACCTTCCCGC |
| *4R MAPT* | GGTGCAGATAATTAATAAGAAGCTGGA | GTGTTTGATATTATCCTTTGAGCCAC |
| *SRSF2* | CTGAGGACGCTATGGATGCCA | GACTTGGACTTGGACCTTCGT |
| *RPL22* | CACGAAGGAGGAGTGACTGG | TGTGGCACACCACTGACATT |
| *UBQLN1* | TGCAGGTCTGAGTAGCTTGG | AACTGTCTCATCAGGTCAGGAT |
